# Using Low-Dimensional Manifolds to Map Relationships Between Dynamic Brain Networks

**DOI:** 10.3389/fnhum.2019.00430

**Published:** 2019-12-10

**Authors:** Mohsen Bahrami, Robert G. Lyday, Ramon Casanova, Jonathan H. Burdette, Sean L. Simpson, Paul J. Laurienti

**Affiliations:** ^1^Laboratory for Complex Brain Networks, Wake Forest School of Medicine, Winston-Salem, NC, United States; ^2^Department of Biomedical Engineering, Virginia Tech—Wake Forest School of Biomedical Engineering and Sciences, Winston-Salem, NC, United States; ^3^Department of Radiology, Wake Forest School of Medicine, Winston-Salem, NC, United States; ^4^Department of Biostatistics and Data Science, Wake Forest School of Medicine, Winston-Salem, NC, United States

**Keywords:** dynamic brain networks, fMRI, connectivity pattern, PCA, t-SNE, embedding

## Abstract

As the field of dynamic brain networks continues to expand, new methods are needed to allow for optimal handling and understanding of this explosion in data. We propose here a novel approach that embeds dynamic brain networks onto a two-dimensional (2D) manifold based on similarities and differences in network organization. Each brain network is represented as a single point on the low dimensional manifold with networks of similar topology being located in close proximity. The rich spatio-temporal information has great potential for visualization, analysis, and interpretation of dynamic brain networks. The fact that each network is represented by a single point makes it possible to switch between the low-dimensional space and the full connectivity of any given brain network. Thus, networks in a specific region of the low-dimensional space can be examined to identify network features, such as the location of brain network hubs or the interconnectivity between brain circuits. In this proof-of-concept manuscript, we show that these low dimensional manifolds contain meaningful information, as they were able to successfully discriminate between cognitive tasks and study populations. This work provides evidence that embedding dynamic brain networks onto low dimensional manifolds has the potential to help us better visualize and understand dynamic brain networks with the hope of gaining a deeper understanding of normal and abnormal brain dynamics.

## Introduction

Brain function emerges from interactions among a massive number of neuronal elements. Although investigations of such complex interactions at a neuronal level still remain beyond the reach of current methodologies, coordinated activities of larger-scale brain regions measured using functional brain imaging methods such as functional magnetic resonance imaging (fMRI) are actively being investigated. Analyses of functional connectivity, which represents the quantified coordination of activity between brain regions, and functional networks generated from thousands of such functional connections, have moved to the forefront of neuroimaging research over the past two decades. There is a growing literature showing that network topology is associated with cognitive and behavioral outcomes (Bressler, 1995; Sporns, 2010; Park and Friston, 2013; Petersen and Sporns, 2015). More recently, it has been reported that functional brain network connectivity patterns fluctuate over short periods of time on the order of seconds (Handwerker et al., 2012; Hutchison et al., 2013; Ma et al., 2014). These *dynamic* fluctuations have been associated with consciousness (Barttfeld et al., 2015; Godwin et al., 2015), learning (Bassett et al., 2011), behavioral responses, and cognitive functions (Cole et al., 2014; Shine et al., 2016), as well as neurodegenerative disorders (Rashid et al., 2016; Zhang et al., 2016). For instance, the integration between specific communities (or modules) of the brain has been shown to increase during a cognitive task (Braun et al., 2015; Finc et al., 2017), and dynamic changes of brain network modular organization have been associated with learning success (Bassett et al., 2011).

Brain dynamics can be conceptualized as transitions between different brain states in response to internal processing and external stimuli (Rabinovich et al., 2012; Nakagawa et al., 2013; Vidaurre et al., 2017). Network neuroscience attempts to model these brain states using whole-brain connectivity patterns. Although each brain state is surely more complex than the connectivity of a given network model, brain network models do effectively capture various normal and abnormal brain processes, as described above. Exceptional challenges facing those interested in dynamic brain networks are generating meaningful visualizations, performing quantitative analyses, and interpreting the vast amounts of data. For instance, for a given study participant, a dynamic network analysis typically yields >100 networks, each with 30,000 or more network connections. To deal with such data, most studies of brain network dynamics have reduced the data to commonly used graph measures, such as node degree and/or modularity (Jones et al., 2012; Shine et al., 2016; Fukushima et al., 2018; Sizemore and Bassett, 2018). Other studies have focused on fluctuations of individual network connections rather than whole-brain dynamics (Elton and Gao, 2015; Hansen et al., 2015; Simony et al., 2016).

Reducing complex brain network dynamics to single graph variables or focusing on individual connections does not take advantage of the wealth of information present in the fluctuations of connectivity across the entire brain network. Representing the whole-brain networks in a low dimensional space that captures similarities and differences in network connectivity would provide a powerful methodology to study network dynamics within and between individual subjects. Ideally, such an embedding procedure would yield a mapping that can be visualized, capitalize on the inherent complexity of the entire brain network, and allow for direct linkages between high dimensional brain networks and low dimensional mapping. Networks with similar topology would be mapped to similar locations in space, and as network topology becomes more and more distinct, the distance between two networks would increase. In such a low dimensional embedding, clusters of similar networks may represent subtle variations in a given brain state, and dynamic transitions between varying connectivity patterns could be examined in time.

There is a growing interest in studying and visualizing brain dynamics in low-dimensional space, but most studies have directly examined the fMRI time series, rather than dynamic networks. Principal components analysis (PCA) has been used to reduce the dimensionality of the raw fMRI data (Shine et al., 2019), and reservoir computing (Venkatesh et al., 2019) has been used to examine temporal state transitions in the raw fMRI data. Both of these studies demonstrated that the patterns of dynamic transitions were unique for distinct task conditions. Taghia et al. (2018) applied a Bayesian switching dynamical systems (BSDS) model to ROI time series from raw fMRI data to identify hidden brain states and state transitions during a working memory task. They showed that the presence/absence of specific (dominant) brain states during the task could predict performance accuracy. They also identified an association between cognition and flexibility in transitioning between hidden brain states.

Several recent studies have mapped brain dynamics on low-dimensional manifolds using methods other than embedding raw fMRI time series. In (Billings et al., 2017), fMRI data were processed with independent components analysis (ICA), and the dynamic brain states were plotted in 2-dimensional (2D) space using the t-distributed stochastic neighbor embedding (t-SNE) algorithm (van der Maaten and Hinton, 2008). Although this study did not directly map individual network dynamics, density maps were created to visualize the portions of this 2D space most commonly occupied under various task conditions. Maps were also created to examine the likelihood of transitioning away from a given point in the 2D space. The data showed that these maps were distinct for different task conditions. Using a maximum entropy model, brain states have been defined as attractors or local minima in the energy landscapes of the brain networks (Watanabe et al., 2014; Ashourvan et al., 2017; Kang et al., 2017). Allen et al. (2014) used a clustering approach to identify functional connectivity patterns which they defined as reoccurring short-term connectivity patterns. Using group ICA components, they first produced a stationary functional network for each subject. Dynamic networks were then generated, and k-means clustering was used to identify the reoccurring connectivity patterns. They demonstrated flexible connections between specific brain regions and identified unexpected functional connectivity patterns involving interactions between large-scale networks. While each of these studies provides interesting insights into brain network dynamics, none of the studies examine dynamic network transitions at the level of the individual subject. In addition, none of the methods used allow for a one-to-one mapping between high-and low-dimensional spaces. In other words, the individual points embedded in low-dimensional space do not represent individual networks in high-dimensional space. If one wants to examine the regional brain circuits and the topology of brain networks occupying particular portions of the low-dimensional space, it is necessary to be able to transition directly from points in low-dimensional space to the actual high-dimensional networks.

In the current study, we propose directly embedding dynamic brain networks into low dimensional space as a means to study network dynamics within and across study participants. This space can be simply generated using various linear and nonlinear embedding techniques, can accommodate large datasets, and can be used for multiple analytical purposes. Dynamic networks embedded into this low-dimensional space maintain their natural temporal sequence. In addition, the similarities or differences between each network and every other network are captured by the spatial locations of the embedded points. Importantly, one can readily examine the original high-dimensional brain network representation for any low dimensional point mapped to this space. Thus, is space preserves information present in high dimensional networks while allowing for visualization, analyses, and interpretation of dynamic connectivity patterns in low dimensional space. In addition, other high dimensional network features, such as modularity or node degree maps, could be added as an additional dimension or even directly integrated into the 2D space (McInnes et al., 2018). We show maps of dynamic brain networks projected into low-dimensional space using linear (PCA) and nonlinear (t-SNE) embedding techniques. As a proof of concept that this low dimensional space contains meaningful information, we classify embedded dynamic networks for different cognitive tasks (rest, 1-back, and 2-back working memory tasks) and different study populations (younger and older adults). We also assessed the spatial clustering of the embedded dynamic networks by condition, population, and individual study participants. We postulate that dynamic brain networks embedded in low dimensional space have the potential to be used for visualization, analyses, and interpretation across different studies.

## Materials and Methods

### Study Participants and Image Collection

Data in this study was collected as part of a prior study examining the effect of the interaction between age and alcohol consumption on brain networks (Mayhugh et al., 2016) in community-dwelling participants. The dataset is comprised of 41 older adults (65–80 years old, sex (M/F) = 22/19) and 22 younger adults (24–35 years old, sex (M/F) = 10/12) that consumed alcohol across a range of consumption levels. This dataset was selected as it contained valuable groups and conditions to assess two types of studies common in the human neuroimaging literature: (1) studies identifying different neural mechanisms underlying various cognitive tasks; and (2) identifying differences between distinct populations or groups. This data set contained multiple study conditions (rest and working memory) as well as two distinct study populations (younger and older adults).

All participants had brain imaging completed on a 3T Siemens Skyra scanner in a single visit. T1-weighted structural data were acquired in the sagittal plane using a single-shot 3D MPRAGE GRAPPA2 sequence (resolution = 0.98 × 0.98 × 1.0 mm, acquisition time: 5 min and 30 s, TR = 2.3 s, TE = 2.99 ms, 192 slices). Resting-state as well as 1-back and 2-back working memory fMRI data (resolution = 3.75 × 3.75 × 5.0 mm) were acquired for each participant using BOLD-contrast images in an echo-planar imaging sequence (acquisition time = 6 min and 20 s, TR = 2.0 s, TE = 25 ms, flip angle = 75°, volumes = 187, slices per volume = 35). Participants were asked to look at a fixation cross during the resting-state scan. For the working memory task, a white letter was presented one at a time on a black background. Participants were asked to respond with either a right (yes) or left (no) finger press to indicate if the letter they were viewing was the same letter that was presented just before (1-back) or two letters before (2-back). For more study details, see Mayhugh et al. (2016).

### Image Preprocessing and Functional Network Generation

Standard image preprocessing was conducted using SPM12^1^. Structural images were segmented into six tissue probability maps: gray matter, white matter, cerebrospinal fluid, bone, soft tissue, and air/background. Gray matter and white matter maps were combined to create a brain tissue map. This image was warped using Advanced Normalization Tools (ANTs; Avants et al., 2011) to Colin space^2^ to match a functional atlas (Shen et al., 2013). The inverse transform produced by ANTs was applied to the functional atlas in order to put the atlas into each subject’s native space. Structural images were then co-registered to each functional image. Resulting transforms were applied to segmentation maps as well as the native space atlas. Other preprocessing of the functional data included: discarding the first 10 volumes to ensure that fMRI signals had achieved equilibrium, slice time correction, realignment to the first volume, band-pass filtering (0.009–0.08 Hz, Power et al., 2012; Yamashita et al., 2018), and regressing six rigid-body transformation parameters that were generated during the alignment process along with average brain tissue signals (gray matter, white matter, cerebrospinal fluid). Functional data were motion-corrected by removing volumes with excessive movement and signal change according to the method in Power et al. (2012).

The brain was parcellated into 268 functional regions (Shen et al., 2013), and whole-brain networks were generated based on these regions. Dynamic networks were created using the sliding window technique. A rectangular window was used that contained 60 volumes (120 s), and a shift size of 1 volume (2 s) was used to generate sequential networks. The shift size of 2 s (one TR) was chosen to maintain the maximum temporal resolution for brain network dynamics. For more detail on the window and shift size choices see Leonardi and Van De Ville (2015) and Mokhtari et al. (2018). We moved the window across the times series, and at each shift, the Pearson’s correlation was computed between the time-series of all ROI pairs. This provided a correlation matrix that represents the functional network at each point (each shift). Weighted, fully connected networks containing positive and negative edges were used for all analyses. Given that there were 187 functional volumes and a window size of 60 volumes, there was a maximum of 127 dynamic networks for any given person/condition. On average, there were 115 networks per person/condition with the number of networks differing across participants due to volumes being removed by the motion correction procedure described above.

### Embedding Dynamic Networks in Low Dimensional Space

**Figure 1:**
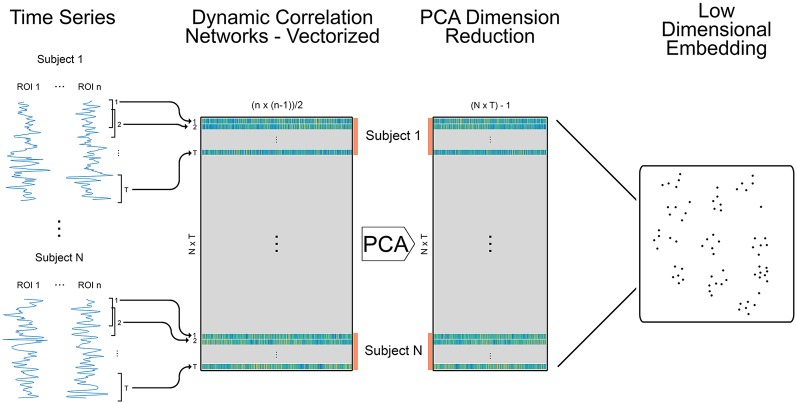
Methods for creating embedded dynamic networks. Pre-processed fMRI scans are converted into time series data representing *n* ROIs and divided into *T* overlapping windows of equal length. Each window is shifted by a step of one time volume from the previous window. For each window, the data is correlated between ROIs to create *T* dynamic networks (Pearson’s correlation matrices) that are symmetric and unthresholded. The process is repeated for *N* subjects. The resulting networks are vectorized retaining only the data above the diagonal and stacked across time and subjects into a single matrix sized (*N × T) × m*, where *(N × T)* is the number of dynamic networks and *m* is the number of edges in each vectorized network (*n* × (*n* − 1)/2). The orange bars denote data for individual subjects. The data is reduced using principal components analysis (PCA) and component weights are formed into a new data matrix. A specified number of component weights are then embedded into 2D space.

**Figure 2:**
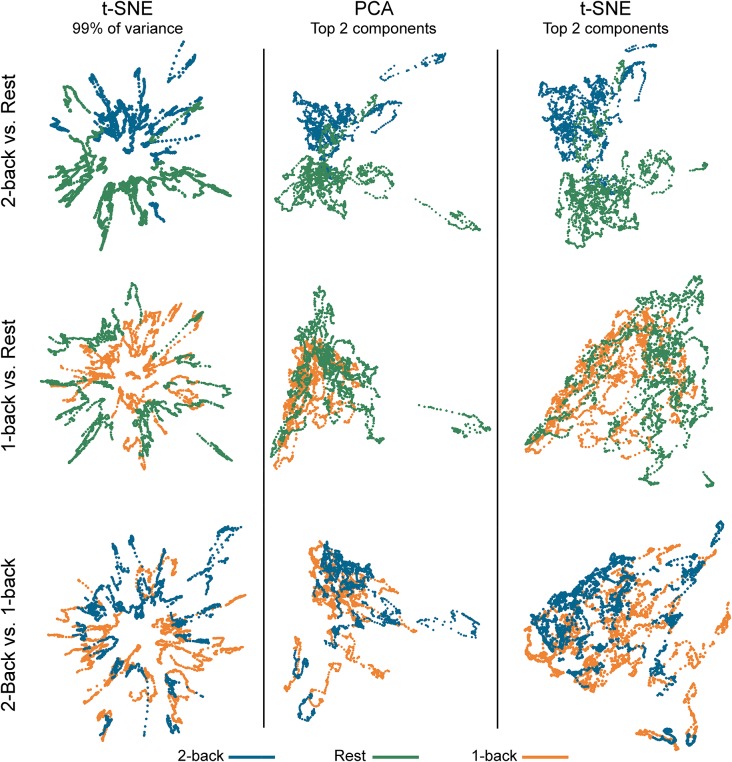
Embedded dynamic brain networks across tasks. Embedded networks are shown for younger adults to contrast 2-back working memory task and rest (top row), 1-back working memory task and rest (middle row), and 2-back and 1-back working memory tasks (bottom row). Each column shows a different 2D embedding method. Although patterns differ between embedding methods, the same trend is apparent across methods: 2-back and rest have the greatest separation, 1-back and rest are less distinct, and 2-back and 1-back have the least separation. The embedded networks are colored by task: 2-back—blue, rest—green, 1-back orange. For both t-distributed stochastic neighbor embedding (t-SNE) datasets, the embedding was repeated 50 times, but only one representative map is shown here.

**Figure 3:**
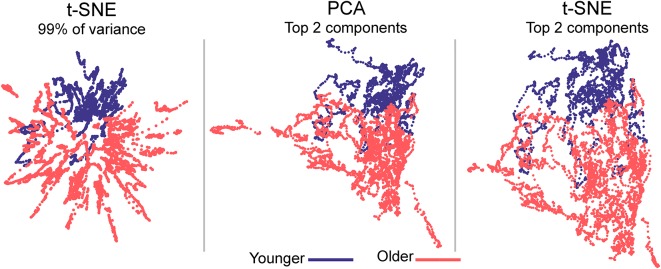
Embedded dynamic brain networks across groups. Embedded networks are shown for younger and older adults during the 1-back working memory task. As with the task-based mapping, the group maps made with t-SNE using 99% of the variance is distinct from the other two mappings. However, the two populations are visibly distinct for all embedding methods. The embedded networks are colored by group: younger—blue and older—red. For both t-SNE datasets, the embedding was repeated 50 times, but only one representative map is shown here.

After the dynamic functional network generation, the networks were reformatted into a combined data structure. Given that the Pearson’s correlation matrices (268 × 268 cells) are symmetric about the diagonal, values above the diagonal were extracted and reshaped into a 1-dimensional vector (35,778 elements) for each network in the dynamic network time series. The vectors were stacked across all time points (115 on average) for a given participant with each row containing the 35,778 correlation values of a single time point. The subjects were then further stacked by adding all rows for each subject to the end of the matrix (Figure 1). The resulting 2D matrix contained 35,778 columns and a row for each and every dynamic network (number of subjects × number of dynamic networks) in the particular analysis. PCA was then used to reduce the dimensionality of this data, yielding a series of components with associated weights. The results of the PCA analysis were specific to the conditions or groups that were included in each comparison. Thus, the individual components, number of components, and the variance captured by each component differed for each analysis. Supplementary Figure S1 contains variance plots for each of the PCA analyses. These figures indicate the number of components needed to capture 99% of the total variance as well as the percent of the variance captured by the first two components. Note that it is vital to ensure that information from test data are not leaked into the training model for the classification portion of this study. It has been demonstrated that dimension reduction using unsupervised learning (as performed here) prior to partitioning the data into training-testing does not result in information leaking from training to testing samples (Hastie et al., 2009). Following the dimension reduction step, the resulting weights were embedded onto a 2D manifold using either linear (PCA) or nonlinear (t-SNE) methods (Figure 1). These 2D representations were used for visualization, classification of study populations and cognitive tasks, and spatial clustering statistics at the group and individual levels. The individual embedded networks were color-coded to indicate study conditions or populations (Figures 2, 3 for example) or to identify individual participants (Supplementary Figures S2–S4).

For linear embedding, the weights for the first two components from PCA, i.e., the two components that captured the most variance, were used for the embedding procedure. This linear embedding method is simply a mapping of the weight of the first PCA component on the X-axis vs. the weight of the second component on the Y-axis. Thus, embedding the data in 2D space limits the analysis to the first two components.

For nonlinear embedding, t-SNE was used (van der Maaten and Hinton, 2008). However, there is no restriction on the method to be used and other methods such as Isomap (Tenenbaum et al., 2000) or UMAP (McInnes et al., 2018) could be used. Two different sets of embedded mappings were created with t-SNE using either the weights from all components needed to capture 99% of the variance or the weights from the first two components. This latter set of mappings was created for direct comparison to the linear embedding. t-SNE is a non-linear machine learning algorithm developed for the reduction and visualization of high-dimensional data. It is an unsupervised algorithm that projects high-dimensional data into a lower space in two main steps. First, a probability distribution over high-dimensional point pairs (fMRI networks in our case) is constructed such that similar (high-dimensional) points get higher probabilities. Then, a probability distribution over low-dimensional data (initially generated randomly or through other data reduction methods) is constructed. The Kullback-Leibler divergence between the two distributions is minimized with respect to the locations of the low-dimensional data to obtain the final low-dimensional points after sufficient number of optimization iterations. The distribution in the high-dimensional space is defined as a standard Gaussian Kernel, while the low-dimensional distribution is defined as a t-distribution. More detail about t-SNE is provided in the Supplementary Material. A MATLAB (2016b) implementation of t-SNE was used for the embedding^3^.

To examine how our network and image analysis parameters affected the embedding results, we investigated additional window sizes, shift sizes, window shapes, and motion correction algorithms on the embedded mappings. Specifically, we embedded dynamic networks generated using 90 and 120 volume windows, 2 and 5 volume shift sizes, and a weighted window (Hamming function). The mappings were all qualitatively similar across the different parameter settings. In addition, we re-processed the raw imaging data and used Automatic Removal of Motion Artifacts (AROMA) methodology to correct for motion artifacts (Pruim et al., 2015) as this method does not delete aberrant image volumes (i.e., volume censoring) and has recently been shown to perform as well as volume censoring (Parkes et al., 2018). The mappings were similar between the two motion correction methodologies. Figures showing the additional embedded networks are all presented in the Supplementary Figures S5–S8.

### Classification Using Low Dimensional Networks

#### Machine Learning Approach

For each classification, we used a support vector machine (SVM) to discriminate between classes of dynamic networks using the spatial coordinates of the dynamic networks in low dimensional space. SVM is a widely-used classifier that finds a maximum-margin hyperplane between the two classes during the training phase (Burges, 1998). A non-linear SVM classifier with a radial basis function (RBF) kernel as implemented in the LIBSVM toolbox for MATLAB (Chang and Lin, 2011) was used to classify the points in 2D space.

#### Evaluation of Classification Performance

To evaluate classification performance a repeated random subsampling (training-testing 68%–32%) was used to ensure that the obtained results can be generalized. The hyper-parameters (c: cost, and g: gamma) were selected using the average across a 5-fold cross-validation procedure (Hastie et al., 2009) with a grid search on hyperplane parameters during training at each subsampling permutation. Due to stochastic components in the t-SNE algorithm, different embedding runs can result in slightly different maps for the same input data. To account for this variability of the t-SNE maps, the t-SNE algorithm was run 50 times, which resulted in 50 datasets for classification. Thus, each classification analysis was repeated 50 times, and the final performance measures were obtained by averaging across these 50 classifications. We used the accuracy, sensitivity, and specificity of the test data set for model performance evaluation. The same evaluation procedure was used for the single PCA datasets obtained from the first two components for each analysis.

#### Analysis

We performed several classification analyses using 2D representations (i.e., 2D points) generated with t-SNE and PCA. Dynamic networks from younger adults were used to classify: (i) rest/2-back working memory task; (ii) rest/1-back working memory task; and (iii) 1-back/2-back working memory tasks. Dynamic networks from the 1-back working memory task were used to classify populations of younger and older adults.

##### Classification of Embedded Task-Based Dynamic Networks

Each dataset was split into training (68%) and test (32%) subsets ensuring that the entire 2D embedding from each participant was either in the training or the test subset. Using the parameters (SVM hyperplane) obtained from the training phase, the label (i.e., −1 or +1) for each data point in the test subset was predicted. In order to classify the dynamic network time series for each participant, the predicted labels for each of the data points from the participant’s embedded dynamic network time series were used. The label with the majority (more than half of the dynamic networks) was used as the label for that participant’s entire dynamic network time series. The predicted and the actual classes of embedded dynamic networks were then used to obtain the accuracy, sensitivity, and the specificity. This process was repeated with 100 permutations of splitting the dataset into the training and test subsets, and the average values across the 100 permutations were used as the performance measures for that classification. This classification procedure was repeated for the 50 t-SNE replications, and the average values of accuracy, sensitivity, and specificity across the 50 classifications were reported as the final classification performance measures. This same approach was used for classifications of PCA datasets (note that for PCA embedding replications are not necessary so a single dataset was used and the average accuracy, sensitivity, and specificity were computed across 100 permutations).

##### Classification of Embedded Population-Based Dynamic Networks

Each dataset was split into training (68%) and test (32%) subsets. To address the unbalanced data with 41 older and 22 younger participants, the same proportion of groups was used in training and test subsets to avoid any potential bias. The similarity between the sensitivity and specificity results indicates that there was no major effect of the population imbalance. Additionally, the class (i.e., group) weights in the SVM optimization process were modified to account for the unbalanced data by choosing a larger weight for the class with smaller number of observations. All data points representing the embedding of each participant were grouped together either in the training or the test subset. We used the same majority vote procedure as the one described above to obtain the predicted class of each embedding. This process was repeated with 100 permutations of splitting the dataset into the training and test subsets, and the average values across the 100 permutations were used as the performance measures for that classification. This classification procedure was repeated for the 50 t-SNE replications, and the average values of accuracy, sensitivity, and specificity across the 50 classifications were reported as the final classification performance measures. This same approach was used for classifications of PCA datasets but with a single embedding.

## Results

### Summary of Methods

In “Visualization of Embedded Dynamic Networks” section, networks projected into the 2D space are described. “Task-Based Dynamic Networks” section details embedded task-based dynamic networks and “Population-Based Dynamic Networks” section details the embedded population-based dynamic networks. The “Mapping Between High and Low Dimensional Networks” section describes the one-to-one mapping of low and high dimensional data. The “Classifying Embedded Dynamic Networks” section describes the classification analyses for both embedded task-based and population-based dynamic networks. Finally, in “Spatial Clustering of Embedded Dynamic Networks” section, we describe the clustering of groups and individuals in the embedded dynamic networks.

### Visualization of Embedded Dynamic Networks

#### Task-Based Dynamic Networks

Figure 2 shows dynamic networks embedded in low dimensional space for the population of young adults with comparisons of 2-back to rest, 1-back to rest, and 2-back to 1- back. The first column in the image shows networks embedded with t-SNE using 99% of the data variance. There is a clear separation between the embedded dynamic networks for the 2-back working memory tasks and rest conditions. This separation is not evident between 1-back working memory task and rest or between 2-back and 1-back working memory tasks. Dynamic networks embedded using the first two PCA components are shown in the middle column of Figure 2. Although the spatial pattern of the networks embedded using PCA is visibly different from networks embedded with t-SNE using 99% of the variance, the overarching pattern across the three task comparisons is quite similar. The separation between the 2-back working memory task and rest is quite clear, but there is extensive overlap between the 1-back and rest as well as between the 2-back and 1-back comparisons. Given that the PCA embedding was based on only a small portion of the total variance, t-SNE was also used to embed networks based only on the first two-component weights for a more direct comparison. The last column of Figure 2 shows these results. It is visually apparent that the shape and spatial distribution of these embedded networks closely resemble those embedded with linear PCA.

#### Population-Based Dynamic Networks

Embedded dynamic networks for younger and older adults performing the 1-back task are shown in Figure 3. The networks embedded using t-SNE with 99% of the variance show clear separation between the younger and older adults. There are notable exceptions with several of the younger adults having networks that overlap with the older adults. It is also evident that the embedded networks from older adults have greater variability in the spatial distribution compared to those from the younger adults. The dynamic networks from individual subjects embedded using the first two PCA components (both PCA and t-SNE embedding) have spatial patterns distinct from those embedded using t-SNE with 99% of the variance. These spatial patterns are comparable to those seen for the between-task comparisons. Despite differences in the appearance of dynamic series from individual subjects, there is a clear separation between the younger and older populations for both embedding methods. The older adults also have greater variability in the spatial distribution of the embedded networks compared to younger adults. All quantitative analyses described below comparing younger and older adults were performed using the 1-back task. However, to be complete, the embedded networks for the 2-back and rest conditions are presented in Supplementary Figure S9.

#### Mapping Between High and Low Dimensional Networks

**Figure 4:**
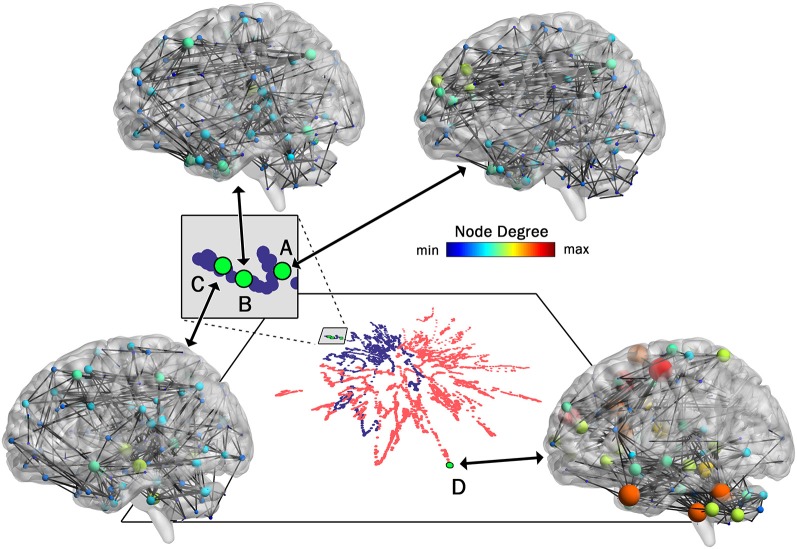
One-to-One mapping of low and high dimensional data. High dimensional networks associated with four sample low dimensional 2D points in the t-SNE embedding (created with 99% of variance) are shown here. Three networks associated with a younger individual (A, B, and C) and one network associated with an older individual (D) are shown. Interestingly, although in the series of dynamic brain networks, network B (85th network) is closer to network A (72th network) in time when compared to network C (117th network), the connectivity of network B is more similar to network C when compared to network A, resulting in closer locations in 2D space. Also, as shown in this figure, all three networks associated with the younger individual are substantially different from the network associated with the older individual. This is clearly captured by the large separating in the low-dimensional space. For visualization purposes, all four networks were thresholded to maintain the strongest 1.5% of connections. Network images were generated using the actual Pearson’s correlation matrices in BrainNet viewer software (Xia et al., 2013).

As pointed out earlier in the introduction, unlike most current methods, the method introduced here allows for a one-to-one mapping between a given network or cluster of networks in low dimensional space and the associated network(s) in high dimensional space. To demonstrate this capability, in Figure 4 we show the mapping between the low and high dimensional data for networks from two of the participants included in Figure 3. This figure shows three networks (A, B, and C) from a younger participant and a single network (D) from an older participant. The figure highlights the low dimensional spatial representation and the high dimensional connectivity of each network. Networks A, B, and C are the 72nd (2:24, min:s), 85th (2:50), and 117th (3:54) networks in the time series of dynamic networks for the younger participant. Network D is the first network of the dynamic network time series for the older participant. As this figure shows, the close proximity in low dimensional space of the three networks from the young individual (A–C) is associated with similar connectivity patterns relative to the network from the older adult (D). For example, the highly connected hub nodes in the motor cortex and cerebellum (red and orange nodes) are present in the older adult, but these nodes have substantially lower degrees in all three networks of the younger adult. Interestingly, the connectivity of network B is more similar to network C than it is to network A (e.g., higher degree hub nodes present in the frontal lobe in network A (green nodes) are not present in network B and C), despite the fact that A and B are closer in time. The pattern is depicted in the low dimensional mapping with B and C being closer together in space than A and B. Graph variables that are frequently used to evaluate brain networks (Bullmore and Sporns, 2009) were computed for each of the individual networks as well as average across the entire dynamic time series for both participants (Table 1). The large spatial separation between the younger and older adult in the low dimensional embedding is supported by consistent differences in the network variables in single networks as well as in the average across the time series. However, the limitation of comparing brain networks using the summary variables is highlighted by the fact that the summary variables did not exhibit consistent patterns between three networks from the younger adult.

**Table 1:** Graph summary variables for networks highlighted in Figure 4.

	Global efficiency	Local efficiency	Assortativity
	**Younger Adult**		
Network A	0.259	0.463	0.707
Network B	0.342	0.442	0.735
Network C	0.274	0.434	0.731
Series Average	0.342	0.450	0.720
	**Older Adult**		
Network D	0.311	0.373	0.691
Series Average	0.315	0.427	0.693

*Note that the “Series Average” is the variable computed and averaged over all networks in that dynamic time series*.

## Classifying Embedded Dynamic Networks

### Task-Based Dynamic Networks

The spatial patterns that are visibly evident in the embedded networks parallel the results obtained when using the embedded networks to classify the task conditions with an SVM (Table 2). For the networks embedded using t-SNE with 99% of the variance, the 2-back/rest conditions were classified with 87.6% accuracy. The classification accuracy fell to 58.8% for the 1-back/rest comparison and classification totally failed for the 2-back/1-back comparison (44.5%). The classification sensitivity and specificity followed similar patterns and are shown in Table 2. Classification accuracy, sensitivity, and specificity of the networks embedded using PCA and t-SNE with two components exhibit the same trends as those for the t-SNE using 99% of the variance. It is notable that the networks embedded using two components tended to have higher classification accuracy than those embedded with t-SNE using 99% of the variance. The first two components capturing the greatest variability happened to contribute the most to discriminating between conditions in our case. The addition of the remaining components to reach 99% of the variance added individual-level variability, and thus, reduced classification performance.

**Table 2:** Classification accuracy for embedded task-based networks.

	Accuracy	Std Dev	Sensitivity	Std Dev	Specificity	Std Dev
	**t-SNE with 99% of the variance**					
2-Back vs. Rest	87.6	6.8	86.4	11.3	88.8	8.5
1-Back vs. Rest	58.8	7.6	67.2	12.7	50.5	12.9
2-Back vs. 1-Back	44.5	8.8	51.8	22.1	37.1	19.6
	**PCA with top 2 components**					
2-Back vs. Rest	90.1	7.9	88.6	12.1	91.4	8.8
1-Back vs. Rest	65.6	11.1	62.2	15.7	69.0	16.2
2-Back vs. 1-Back	52.4	12.4	56.9	18.9	47.9	18.3
	**t-SNE with top 2 components**					
2-Back vs. Rest	90.0	7.8	88.9	11.8	91.1	9.0
1-Back vs. Rest	64.8	10.6	57.9	16.4	71.7	14.5
2-Back vs. 1-Back	50.3	11.9	56.1	22.7	44.4	21.7

*Each measure is an average of over 100 permutations of the SVM training. The same permutations were used for all embedding methods. For both t-SNE classifications, each permutation is averaged across the 50 embedding replications as well*.

**Figure 5:**
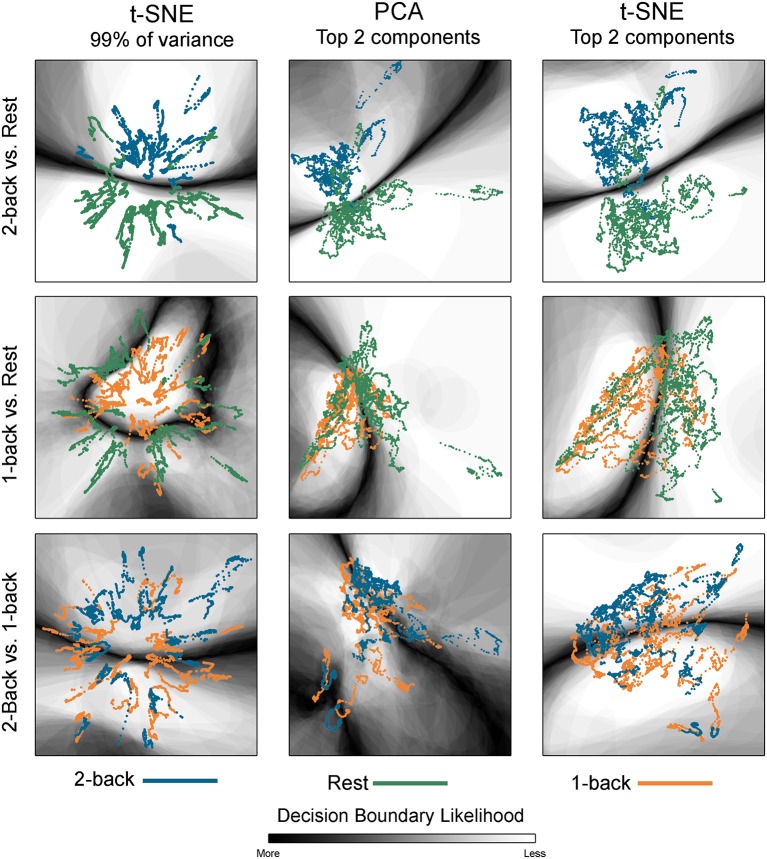
Classification boundaries for embedded task-based networks. Boundaries for younger adults comparing 2-back working memory task and rest (top row), 1-back working memory and rest (middle row), and 2-back and 1-back working memory tasks (bottom row). The dynamic networks are overlaid on the classification boundaries for visualization purposes. Each column shows a different 2D embedding method. The decision boundaries generated from 100 permutations of classifier training/testing were averaged to produce these maps. The darker the background shading the more likely a boundary was present in that location across the 100 permutations. Dark-light interfaces separate networks from different conditions. The probability of being consistently classified across permutations is higher for networks located in the lighter regions. For the 2-back/rest comparisons the boundary makes a fairly clear separation between networks from the two conditions for all embedding methods. For the 1-back/rest comparison, the conditions are not as well separated and the boundaries are less distinct. For the 2-back/1-back comparisons the boundaries are much less distinct and are widely spread across the space. The classification accuracy closely parallels the distinctness of the boundaries. For both t-SNE datasets, only one representative map of the decision boundary (from the 50) is shown here.

The classification boundaries are visualized in Figure 5. This image shows the average boundary across the 100 training/testing permutations for each classification. The darker shading is associated with a higher consistency of boundary location. In areas where there is clear separation between networks for the two conditions, the boundary is more discrete and darker. In areas where the separation between conditions is less clear, the boundary thickens and lightens, indicating that the dynamic networks were variably classified across the 100 permutations. Note that for the 2-back/rest maps, the primary boundary was fairly discrete and was located between the task-specific networks. The majority of the remaining space was lightly shaded, indicating that it is unlikely that a boundary would be found in those areas. There were some areas where networks from both conditions co-existed and boundaries were present on some of the permutations, but the likelihood was relatively low as indicated by the light shading (for example, middle left region for the tSNE99 map). For the other classifications with relatively lower accuracy, the boundary becomes less discrete.

### Population-Based Dynamic Networks

**Figure 6:**
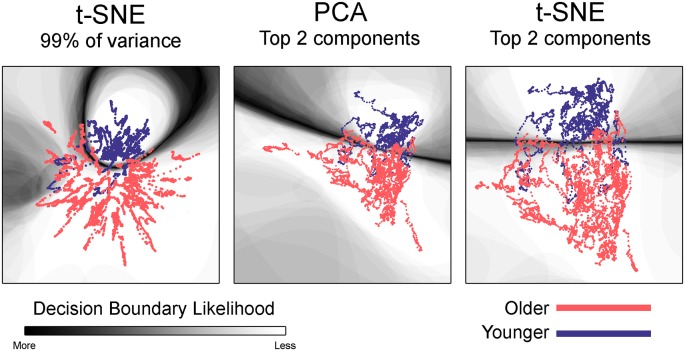
Classification boundaries for embedded group-based networks. Boundaries for classifying younger vs. older adults during the 1-back working memory task. Embedding methods are labeled above each mapping. For all embedding methods, the boundary is fairly distinct and clearly separates the study groups. The dynamic networks are overlaid on the classification boundaries for visualization purposes. See Figure 5 for more details concerning figure interpretation. For both t-SNE datasets, only one representative map of the decision boundary (from the 50) is shown here.

Given the visibly clear separation between the embedded networks for younger and older adults, it is not surprising that classification accuracy was quite high for the group embedding maps. Classification accuracy was 88.2% for networks embedded using t-SNE with 99% of the variance, 88.5% for the networks embedded with PCA, and 88.9% for networks embedded using t-SNE with two components. Table 3 presents the average classification accuracy, sensitivity, and specificity across all three embedding methods. The classification boundaries are visualized in Figure 6. As with Figure 5, the image shows the average boundary across the 100 training/testing permutations with darker shading being associated with higher boundary location consistency. The figure shows clear boundaries between younger and older adults for all three embedding methods. The classification boundaries for embedded networks of the younger and older participants during the rest and 2-back conditions are shown in Supplementary Figure S10.

**Table 3:** Classification accuracy for embedded group-based networks.

	Accuracy	Std Dev	Sensitivity	Std Dev	Specificity	Std Dev
	**t-SNE with 99% of the variance**					
Younger vs. Older	88.2	5.3	85.6	11.0	89.6	7.2
	**PCA with top 2 components**					
Younger vs. Older	88.5	6.7	81.9	13.7	92.0	8.1
	**t-SNE with top 2 components**					
Younger vs. Older	88.9	6.6	83.1	13.7	92.0	8.1

*Each measure is an average of over 100 permutations of the SVM training. The same permutations were used for all embedding methods. For both t-SNE classifications, each permutation is averaged across the 50 embedding replications as well*.

## Spatial Clustering of Embedded Dynamic Networks

The classification results presented above indicate that there is distinct spatial clustering for many of the dynamic embedding maps, but the classification methodology is not intended for quantifying the spatial clustering. To determine how well the data clustered by task, group or by individual participant, the average distance between embedded dynamic networks was assessed. The distance between each pair of dynamic networks was computed as the average of Euclidean distance between all pairs of 2D points from one embedding and all 2D points from the other embedding. The distance within tasks/groups was compared to the distance between tasks/groups to determine how tightly the participants clustered together based on the task or their study population. A permutation test was used to determine if the clustering was significantly greater than expected by random chance.

Clustering at the individual level indicates that the dynamic embedding for that person is distinct from other people, even if they are part of a cluster based on task or group. In order for an embedding method to simultaneous capture population and individual level clustering, it must maintain multiscale properties in the reduced dimensional space. To evaluate the clustering at the individual level, the distance within the individual participants’ embedded dynamic networks was compared to the distance between individuals, and a permutation statistic was used to assess significance. Here, the distance within each participant’s embedding was computed as the average of Euclidean distance among all pairs of 2D points of that same embedding, and the distance between two series of dynamic networks was computed as the average of Euclidean distance between all pairs of 2D points from one embedded series and all 2D points from the other embedded series.

### Task/Group Clustering

Tables s 4, 5 show the ratio of the within task/group to the between task/group spatial distances, and statistics for each of the task/group comparisons based on the embedding method. Smaller ratios indicate that the clustering within tasks/groups was higher than between tasks/groups. As the within task/group clustering decreases, the ratio approaches one (1). As expected based on the visualization and the classification results, clustering of the task-based networks was greatest for the 2-back/rest comparison for all methods. Clustering was highly significant for all embedding methods. For the 1-back/rest comparison clustering was substantially lower than for the 2-back/rest comparison but all methods exhibited significant clustering. The 2-back/1-back comparison did not show significant clustering for any of the embedding methods, consistent with the visualization and classification results.

**Table 4:** Clustering of embedded task-based networks.

	Within/Between	Std Dev	*p*-value
	**t-SNE with 99% of the variance**		
2-Back vs. Rest	0.7263	0.0178	0.0009
1-Back vs. Rest	0.9662	0.0210	0.0186
2-Back vs. 1-Back	1.0055	0.0110	0.1613
	**PCA with top 2 components**		
2-Back vs. Rest	0.6728	-	0.0043
1-Back vs. Rest	0.9361	-	0.0068
2-Back vs. 1-Back	1.0011	-	0.0992
	**t-SNE with top 2 components**		
2-Back vs. Rest	0.6117	<0.0001	0.0043
1-Back vs. Rest	0.9266	0.0032	0.0072
2-Back vs. 1-Back	0.9937	<0.0001	0.0829

*The ratio of the embedding distance within tasks vs. the embedding distance between tasks. For the t-SNE embedding, each measure is an average of over 50 unique replications. For PCA there is only one possible embedding so there is no variance associated with the measures. Significant clustering was identified using *P*-values that were determined for each embedding using a permutation test. The average *p*-values over the 50 embedding replications are shown for t-SNE*.

**Table 5:** Clustering of embedded group-based networks.

	Within/Between	Std Dev	*p*-value
	**t-SNE with 99% of the variance**		
Younger vs. Older	0.6931	0.0165	<0.0001
	**PCA with top 2 components**		
Younger vs. Older	0.6545	-	<0.0001
	**t-SNE with top 2 components**		
Younger vs. Older	0.6238	0.0002	<0.0001

*The ratio of the embedding distance within groups vs. the embedding distance between groups. For the t-SNE embedding, each measure is an average of over 50 unique replications. For PCA there is only one possible embedding so there is no variance associated with the measures. Significant clustering was identified using *P*-values that were determined for each embedding using a permutation test. The average *p*-values over the 50 embedding replications are shown for t-SNE*.

For the comparison of younger vs. older adults, all three embedding methods showed significant clustering by group. Statistical comparisons were made between the embedding methods to identify differences in clustering. Two-sample *t*-tests were used to compare the two t-SNE embedding methods. One-sample tests were used to compare PCA to the t-SNE methods. All comparisons were significant with *p* < 0.001 after correcting for multiple comparisons. The networks embedded using t-SNE with two components always exhibiting the highest clustering, followed by PCA, and then t-SNE with 99% of the variance.

### Individual Participant Clustering

Tables s 6, 7 show the ratio of the spatial distances within individuals relative to between individuals across tasks and groups, respectively. Smaller ratios indicate higher clustering of embedded dynamic networks at the individual level, i.e., the embedded series of dynamic networks for each individual is spatially distinct from other individuals. The embedded dynamic networks were significantly clustered by individual across all tasks/conditions for all embedding methods. All comparisons between the embedding methods were highly significant (*p* < 0.001) after correcting for multiple comparisons. Unlike the task/group level clustering, individual clustering was always highest for the networks embedded using t-SNE with 99% of the variance, followed by PCA, and then t-SNE with two components. Two-sample *t*-tests were used to compare the two t-SNE embedding methods. One-sample tests were used to compare PCA to the t-SNE methods.

**Table 6:** Clustering of embedded individual participant networks across tasks.

	Within/Between	Std Dev	*p*-value
	**t-SNE with 99% of the variance**		
2-Back vs. Rest	0.1738	0.0027	<0.0001
1-Back vs. Rest	0.1897	0.0032	<0.0001
2-Back vs. 1-Back	0.1835	0.0022	<0.0001
	**PCA with top 2 components**		
2-Back vs. Rest	0.2371	-	<0.0001
1-Back vs. Rest	0.2862	-	<0.0001
2-Back vs. 1-Back	0.2234	-	<0.0001
	**t-SNE with top 2 components**		
2-Back vs. Rest	0.2386	<0.0001	<0.0001
1-Back vs. Rest	0.2962	0.0004	<0.0001
2-Back vs. 1-Back	0.2217	<0.0001	<0.0001

*The ratio of the embedding distance within participants vs. the embedding distance between participants. For the t-SNE embedding, each measure is an average of over 50 unique replications. For PCA there is only one possible embedding so there is no variance associated with the measures. Significant clustering was identified using *P*-values that were determined for each embedding using a permutation test. The average *p*-values over the 50 embedding replications are shown for t-SNE*.

**Table 7:** Clustering of embedded individual participant networks across groups.

	Within/Between	Std Dev	*p*-value
	**t-SNE with 99% of variance**		
Younger vs. Older	0.1932	0.0032	<0.0001
	**PCA with top 2 components**		
Younger vs. Older	0.2331	-	<0.0001
	**t-SNE with top 2 components**		
Younger vs. Older	0.2248	0.0018	<0.0001

*The ratio of the embedding distance within participants vs. the embedding distance between participants. For the t-SNE embedding, each measure is an average of over 50 unique replications. For PCA there is only one possible embedding so there is no variance associated with the measures. Significant clustering was identified using *P*-values that were determined for each embedding using a permutation test. The average *p*-values over the 50 embedding replications are shown for t-SNE*.

## Discussion

The current study was designed to determine if embedding dynamic functional brain networks on low-dimensional manifolds can help resolve current challenges associated with visualizing, analyzing and interpreting these networks. As a proof-of-concept we utilized linear plots of PCA components and nonlinear transformations using t-SNE to embed dynamic functional brain networks onto a 2D manifold. This method facilitated visualization and maintained a one-to-one mapping between networks in low- and high-dimensional space. Representations of the networks in the low dimensional space were used to examine the spatial patterns associated with various task conditions (rest, 1-back, and 2-back) and study populations (younger and older adults). We demonstrated that the low-dimensional network representations contained meaningful information sufficient to discriminate between these different task conditions and study populations.

Our analyses in the low dimensional space showed that the separation between dynamic networks across task conditions was greatest for the most distinct task conditions (2-back vs. rest) and for the population comparisons (younger vs. older adults). For conditions that were less distinct (1-back vs. rest and 2-back vs. 1-back), the separation between groups of embedded networks decreased. This was visually apparent and confirmed by quantitative analyses. As the task conditions became more similar and the low dimensional representations merged, the individual participant variability began to dominate. This is demonstrated in Supplementary Figures S2–S4 where the mapped dynamic networks are colored by the individual subject. These figures show that the dynamic networks for an individual subject tend to be furthest apart for the 2-back/rest mapping, regardless of the embedding method. The pair of dynamic networks for individual subjects is closer together for the 1-back/rest. For the 2-back/1-back mapping, the dynamic networks for most subjects are either adjacent to each other or are actually overlapping. The average distance between the low dimensional representations (Supplementary Table S1) showed that the 2-back/rest separation was significantly larger than separation between for the 1-back/rest or the 2-back/1-back mappings for all methods. Although the 1-back/rest separation tended to be greater than the 2-back/1-back separation, this was only significant for t-SNE using two components. Results of the multivariate analysis of variance (MANOVA) used to compare the distances are in Supplementary Table S2.

The low-dimensional visualizations generated using 99% of the data variance exhibited a somewhat “star burst” appearance while those using just two components were more globular. There is currently no* a priori* information to help explain their distinct visual appearance. These networks embedded using 99% of the variance had lower clustering at the task/group level, and classification tended to have lower accuracy, sensitivity, and specificity compared to the maps based on two-components. However, the networks embedded using 99% of the data variance had higher spatial clustering at the individual level compared to those based on two PCA components. Thus, limiting the transformations to these components enhanced differences between the task/group. When the networks are embedded using components that captured 99% of the variance, the low dimensional representations are more likely to be influenced by individual variability captured by the additional components. It is possible that embedding networks to specifically target group, condition, or individual differences could be enhanced if other variables such as population labels, task performance, or individual phenotypic variables (e.g., sex, IQ, age, etc.) are included. This may be achieved by using regression techniques (e.g., by modifying regression tools provided in Bahrami et al., 2019a) for dynamic network analyses) or by capitalizing on new developments in the field of manifold learning, such as Uniform Manifold Approximation and Projection—UMAP (McInnes et al., 2018) which extends the capabilities already available in t-SNE. Future studies can investigate these possibilities.

In addition to using embedded dynamic networks for discriminating between various conditions or populations, the low-dimensional embedding has the potential to be used in mechanistic studies of brain dynamics. The growing interest in studies of brain dynamics is built around the premise that brain states can be modeled using patterns of brain activity or brain connectivity (Hutchison et al., 2013; Khambhati et al., 2018). As noted in the introduction, there is no ideal model of brain states. Measures of brain activity based on fMRI have suggested that specific regions of the brain play crucial roles in brain state transitions (Gu et al., 2017, 2018). Using a graph-based analysis of fMRI signal amplitude, it has been shown that higher flexibility of transitioning between brain states was associated with learning progress (Reddy et al., 2018) and with executive performance differences between children and young adults (Medaglia et al., 2018). Compared to direct measures of fMRI signal amplitudes, brain networks contain a wealth of complex information that may better represent brain states (Bullmore and Sporns, 2009; Ashourvan et al., 2017). Unfortunately, the dynamic brain networks are large and complex making it difficult to identify and interpret meaningful dynamic patterns.

The current study was designed to address this challenge by embedding dynamic networks on a low-dimensional manifold. In a comparable study, Billings et al. (2017) defined brain states using networks obtained from computing the correlation between pairwise state vectors generated with ICA. These states were mapped into a 2D t-SNE space for all study subjects, and density plots were generated to map the probability of existing in specific brain states. However, the individual participant and temporal aspects of the data were not captured, and it was not possible to directly transition from the low-dimensional space back to individual brain networks. Much work remains to be done in order to determine the full potential of using low-dimensional manifolds to study dynamic functional brain networks. The major contributions of the current study are that dynamic network series were embedded, visualized, and analyzed for individual study participants. Our work demonstrates that it is possible to deal with large amounts of information contained in dynamic networks by representing them in a low dimensional space. The representation in this space made it possible to visualize and quantitatively compare the similarities and differences of dynamic networks within and between individuals. Since there is a one-to-one mapping between the low- and high-dimensional spaces, key networks can be mapped back to brain space for mechanistic studies. Cognitive processes may map to specific portions of this space such that the location of the embedded network is indicative of the underlying cognitive process and critical brain circuits can be discovered from the associated high-dimensional brain networks. Another potential use for this embedding method in cognitive neuroscience is real-time fMRI, where visual inspection of results can be very important. It is also possible that embedded dynamic networks could be used in understanding brain disorders or to assess the effectiveness of treatments. For example, low-dimensional network representations could be examined in alcohol or substance use disorder to determine if the cue-induced craving is associated with specific portions of the embedding space. The brain circuits that are mapped to the dysfunctional portions of the space can be examined to identify underlying neural mechanisms.

The current study is not without weaknesses. First, the total number of subjects used (22 in the paired task comparisons and 63 population comparisons) was relatively small. The slightly high standard deviation of classification results is most likely due to this small sample size. We chose to use a dataset that we had in house because our prior work (Mayhugh et al., 2016; Bahrami et al., 2019b) had demonstrated network differences for both the task and group, albeit for static networks. This smaller dataset also avoided the growth in the computational intensity of this methodology associated with increasing the number of networks. While it is possible to deal with very large datasets with algorithm optimization and cluster computing, that was beyond the scope for this proof-of-concept study. We recognize that this methodology will need to be replicated in larger study populations—a goal for future work. Another weakness of this study is that we did not directly compare our approach to other representations created from fMRI time series. For example, representations generated from the fMRI time series using reservoir computing have been shown to discriminate between 2-back and 0-back task conditions with 77–81% accuracy (Venkatesh et al., 2019). Nevertheless, we feel that the brain networks reveal important neural processes that are captured only by examining the relationships between brain regions. Finally, we used motion scrubbing to address head motion artifacts. This method results in removing some volumes, and, thus, participants had different numbers of fMRI scans that went into the analysis. This does not create large temporal gaps in the dynamic network time series as each dynamic network was made from a 120 s window. However, we acknowledge that this procedure likely induces temporal smoothing, and future work is needed to examine how various motion correction methods such as AROMA (Pruim et al., 2015) affect the results.

To our knowledge, this is the first study to demonstrate the promise of embedding dynamic functional brain networks into 2D space to visualize and analyze these complex datasets at the individual and group level. Both linear and nonlinear embedding methods proved useful with each method having its own strengths and weaknesses. The potential utility of examining the spatial location of embedded dynamic networks include, but are not limited to: comparing connectivity patterns from various conditions or study populations, identifying a hierarchy in transitioning between connectivity patterns, determining if disorders are associated with transitioning (or not transitioning) between specific connectivity patterns, classification or prediction in different treatment or disease populations, and relating various phenotypic characteristics (e.g., IQ, BMI, etc.) to unique network dynamics. Our approach has the potential to serve as a cross-study method for representing, analyzing, and interpreting dynamic brain networks. This could ultimately provide a standard space for projecting brain networks where the 1:1 mapping between high and low dimensional spaces is maintained. Thus, quantitative analyses and visualization may be performed on the low dimensional data, and mechanistic hypotheses focused on critical brain regions or circuits can still be assessed in the high dimensional brain networks.

## Data Availability Statement

The datasets generated for this study are available on request to the corresponding author.

## Ethics Statement

The studies involving human participants were reviewed and approved by Wake Forest School of Medicine Institutional Review Board. The patients/participants provided their written informed consent to participate in this study.

## Author Contributions

MB conceptualized the idea and introduced minor modifications to it, performed the classification and statistical analyses and writing. PL introduced the idea, supervised the findings and writing. RL performed data preprocessing, t-SNE and PCA map generation computations, presented important suggestions in developing the idea and writing. RC provided expertise relating to the t-SNE algorithm, verified the analytical methods and writing. JB introduced the idea and writing. SS performed the statistical tests, verified statistical test results and writing. All authors discussed the results and contributed to the final manuscript.

## Conflict of Interest

The authors declare that the research was conducted in the absence of any commercial or financial relationships that could be construed as a potential conflict of interest.

## Footnotes

^1^www.fil.ion.ucl.ac.uk/spm/

^2^http://www.bic.mni.mcgill.ca/ServicesAtlases/Colin27

^3^https://lvdmaaten.github.io/tsne/code/tSNE_matlab.zip
