# Supplementary material for: Using Low-Dimensional Manifolds to Map Relationships Between Dynamic Brain Networks
Source: Front Hum Neurosci. 2019 Dec 10;13:430. doi: 10.3389/fnhum.2019.00430 (PMC6914694; doi:10.3389/fnhum.2019.00430)
Supplement: Supplementary file 1 [file Data_Sheet_1.docx]

# T-distributional Stochastic Neighboring Embedding (t-SNE)

t-SNE is a non-linear machine learning algorithm developed for visualization of high-dimensional data. It is an unsupervised algorithm that projects high-dimensional data into a lower space in two main steps. First, a probability distribution over high-dimensional point pairs is constructed such that similar (high-dimensional) points get higher probabilities. Then, a probability distribution over low-dimensional data is constructed, and the Kullback-Leibler divergence between the two distributions (Eq. 3) is minimized with respect to the locations of the low-dimensional data to obtain the final low-dimensional points after sufficient number of optimization iterations. The distribution in the high-dimensional space is defined as a standard Gaussian Kernel (Eq. 1), while the low-dimensional distribution is defined as a t-distribution (Eq. 2).

$p_{ij}=\exp\frac{\exp\left(-\left\|x_{i}-x_{j}\right\|^{2}/2\sigma^{2}\right)^{-1}}{\sum_{k\neq l} \exp\left(-\left\|x_{k}-x_{l}\right\|^{2}/2\sigma^{2}\right)^{-1}}$ (1)

Where $p_{ij}$is the similarity between the brain networks $x_{i}$ and $x_{j}$ in the high-dimensional space $R^{D}$, In similar fashion, similarities between low dimensional representations of the brain networks $y\in R^{d}$ are defined as

$q_{ij}=\frac{\left(1+\left\|y_{i}-y_{j}\right\|^{2}\right)^{-1}}{\sum_{k\neq l} \left(1+\left\|y_{k}-y_{l}\right\|^{2}\right)^{-1}}$ (2)

The similarity measure in the low dimensional space is based on heavy tailed Student-t distribution to account for the large difference of volume between the high- and low-dimensional spaces. The representation $y_{1}...y_{N}\in R^{d}$ is learned by minimizing the Kullback-Leibler divergence between the two distributions:

$KL\left(P\text{||}Q\right)=\sum_{i\neq j} p_{ij}\log\left(\frac{p{}_{ij}}{q_{ij}}\right)$ (3)

Optimization of Eq. 3 is a complex process based on the gradient descent method (GDM). The main parameters and the values we used are: 1) perplexity which is a smooth measure of the effective number of neighbors = 85% of the samples; 2) learning rate =1000; 3) initial momentum =0.5; 4) final momentum=0.8 and 5) momentum switch iteration=200. More detail about these parameters, the optimization procedures and the t-SNE methods can be found in the references provided in the main text. In our study t-SNE constructs a map in a low dimensional space in which each point corresponds to one fMRI brain network by defining a similar distribution over pairs of points and minimizing the divergence between the two distributions. It defines pairwise similarities between fMRI brain networks that have already been reduced to PCA components $x\in R^{D}$(where D is either two (2) or the number of components capturing 99% variability of fMRI brain networks).

# Supplementary Figures and Tables


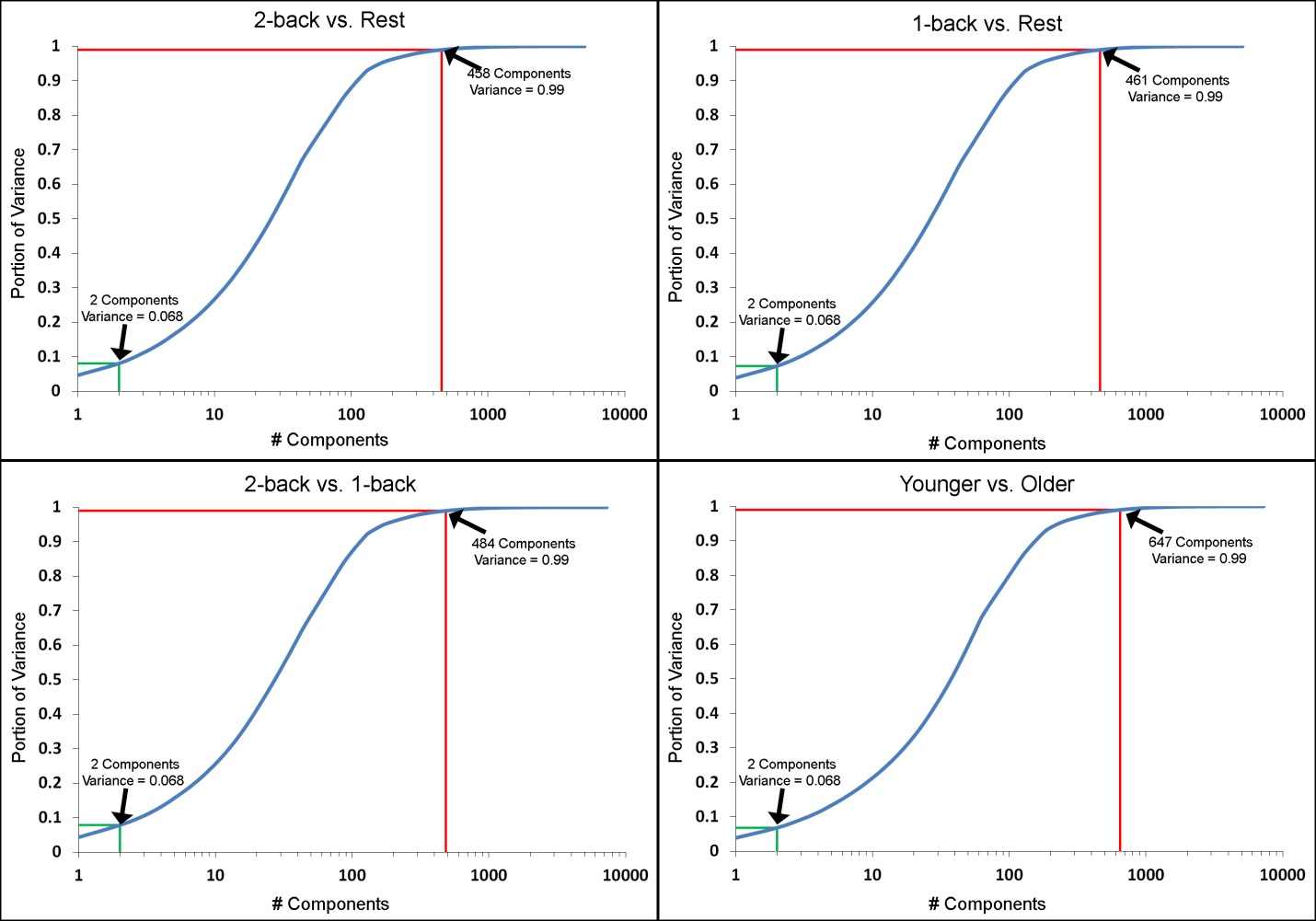


**Supplemental Figure 1.** **PCA component-variance plots**. The figures show the cumulative proportion of variance captured by the PCA components for each of the task/group comparisons. The number of components needed to capture 99% of the variance is indicated by the red lines. The proportion of the variance captured by the top two components is indicated by the green lines.

| 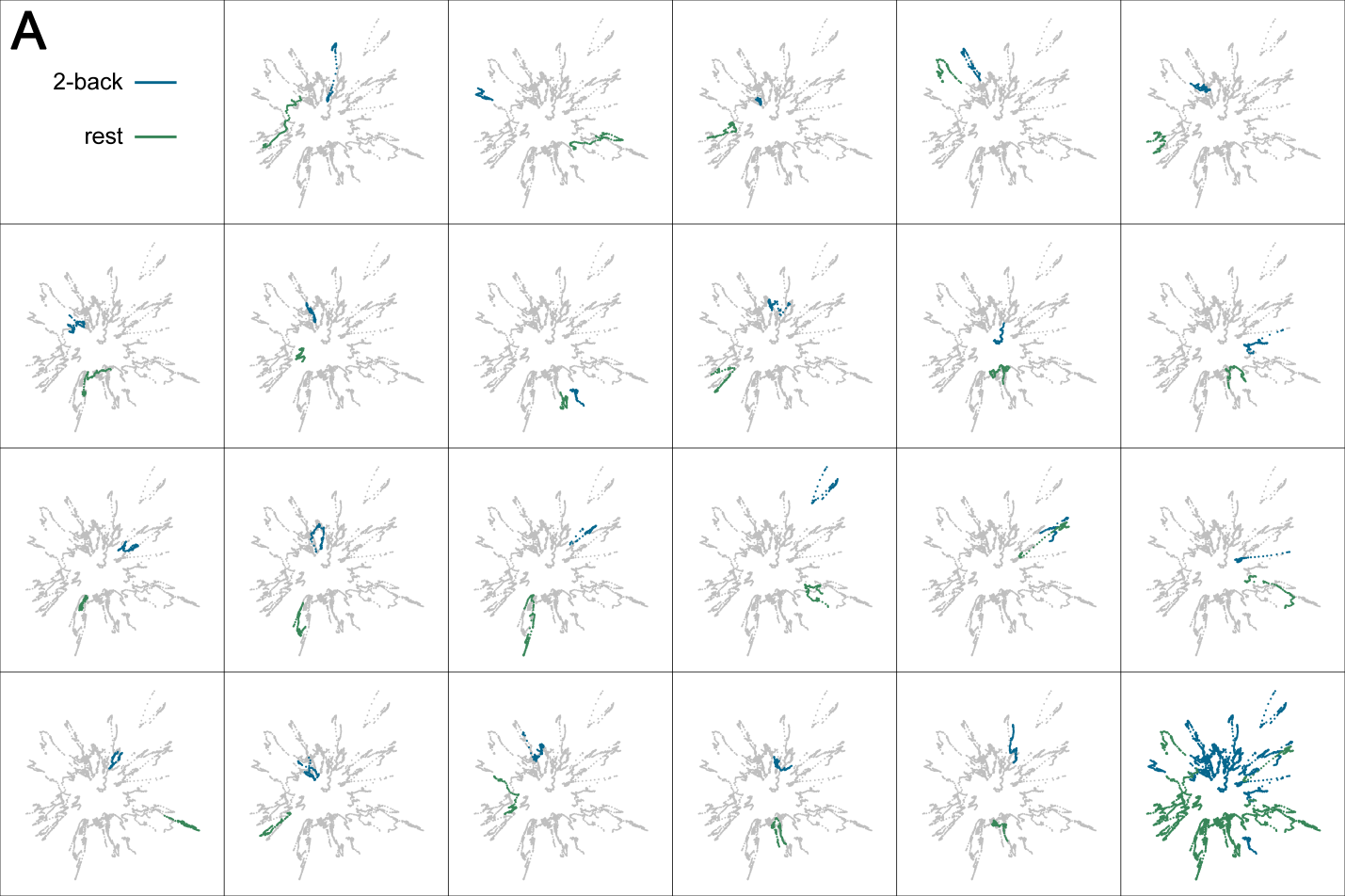 |
| --- |
| 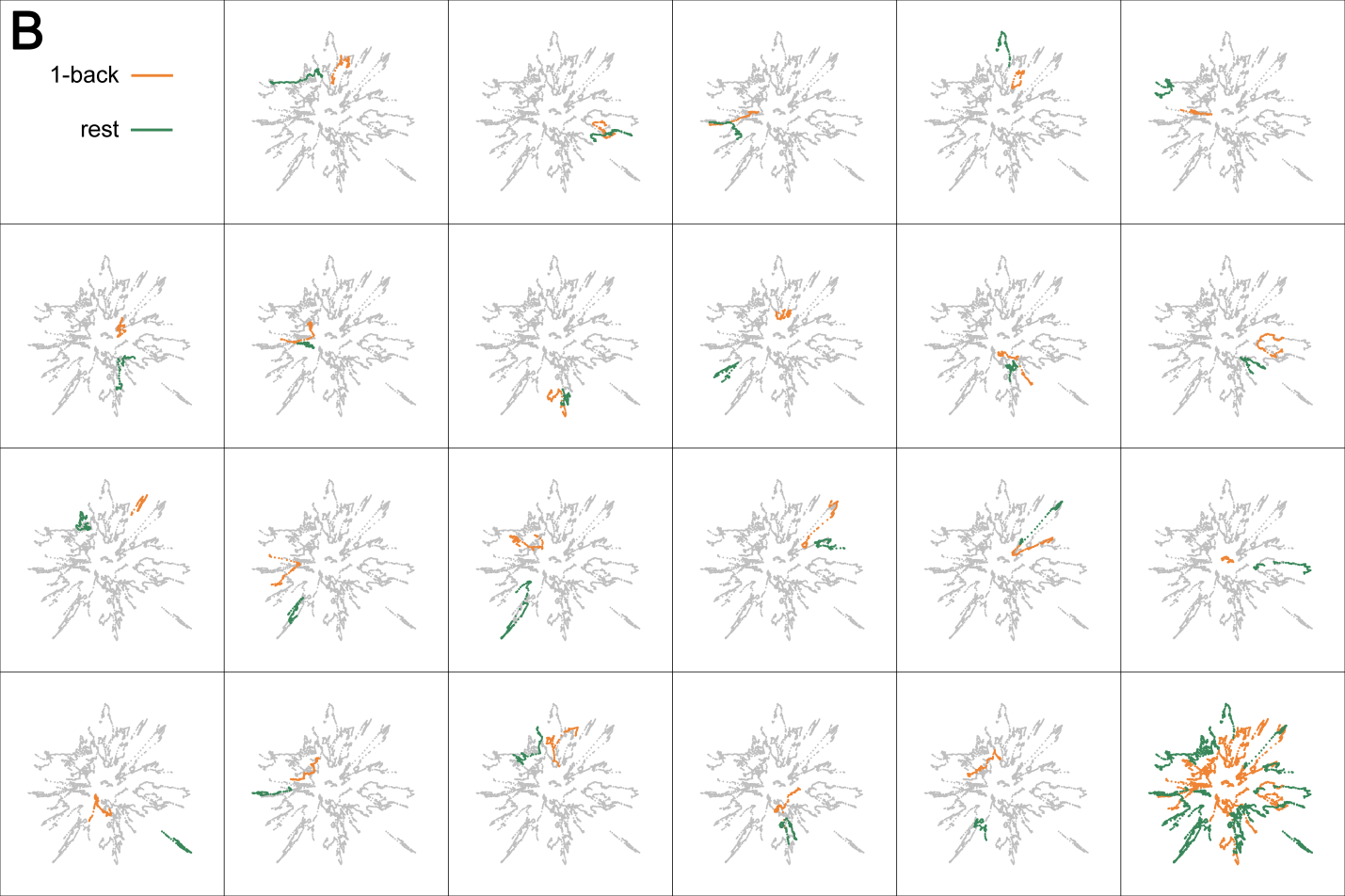 |

| 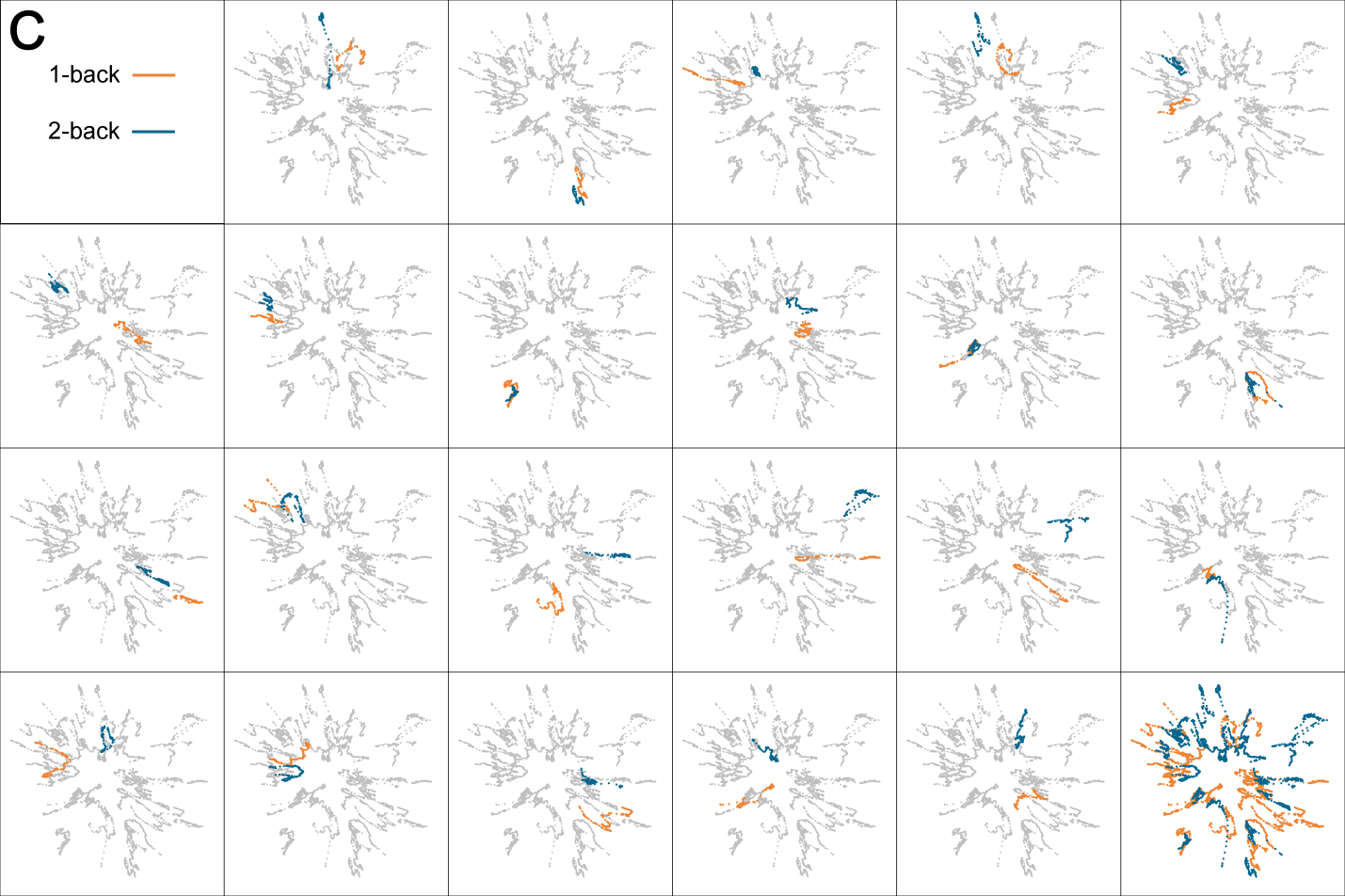 |
| --- |

**Supplemental Figure 2.** **Task-based embedded** **dynamic networks colored by individual participant generated with t-SNE using 99% of the variance**. Each of the 22 individual participants is represented in a separate panel with their embedded networks colored by task for **(A)** 2-back/rest, **(B)** 1-back/rest, and **(C)** 2-back/1-back. In each of the participant-specific panels all other participants are gray. The bottom right corner of each figure shows all embedded networks colored as shown in the top left corner.

| 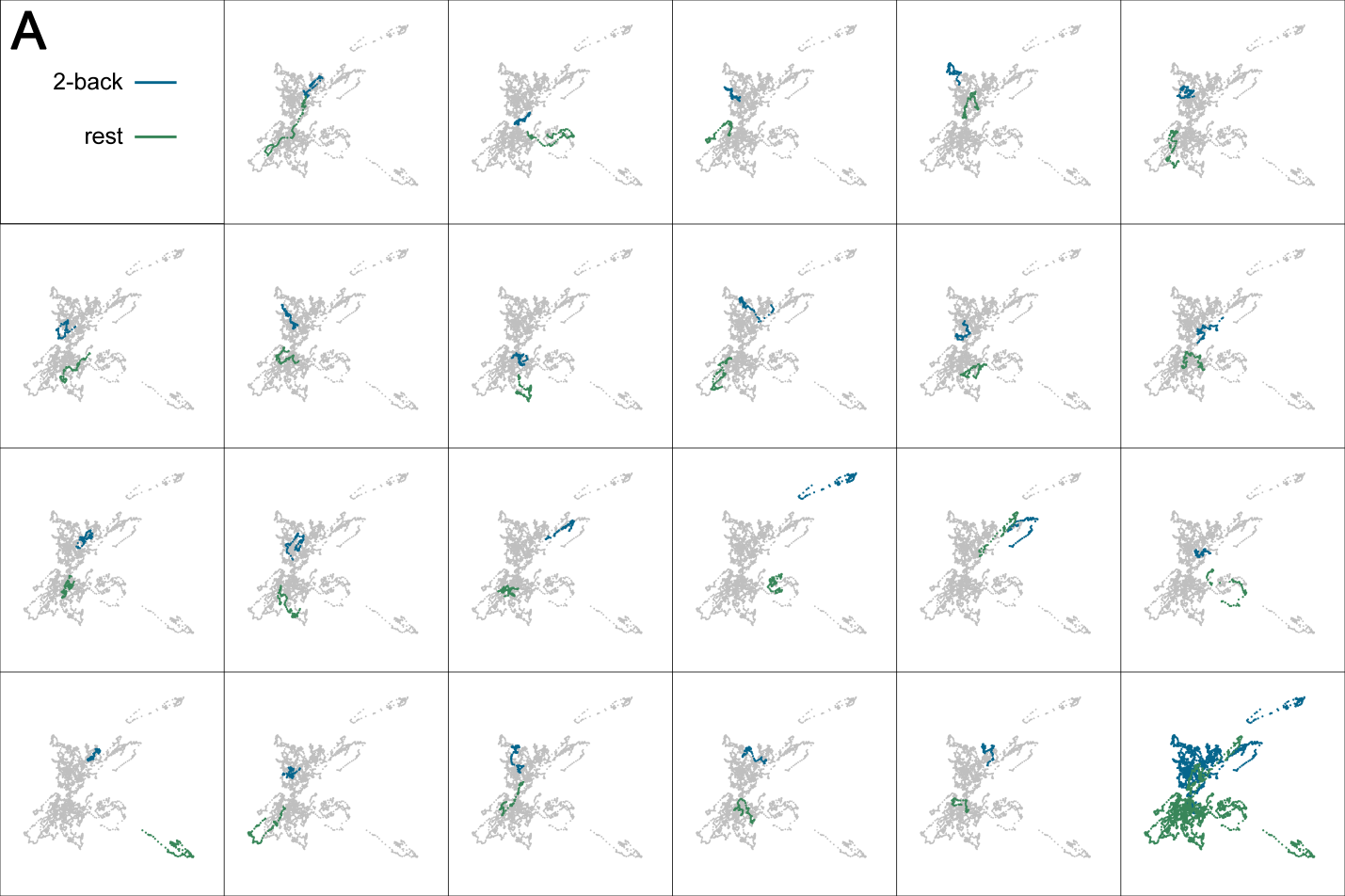 |
| --- |
| 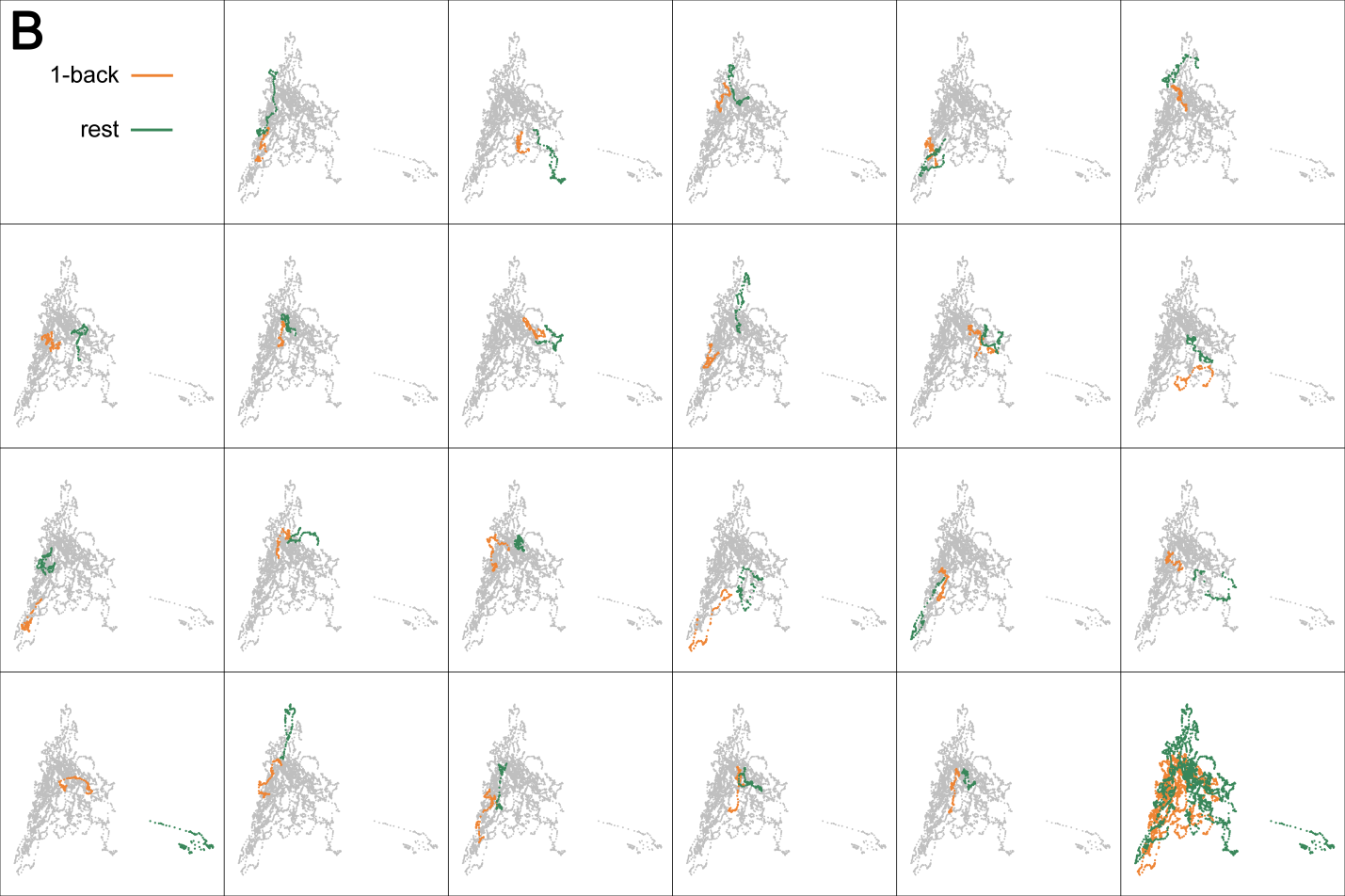 |

| 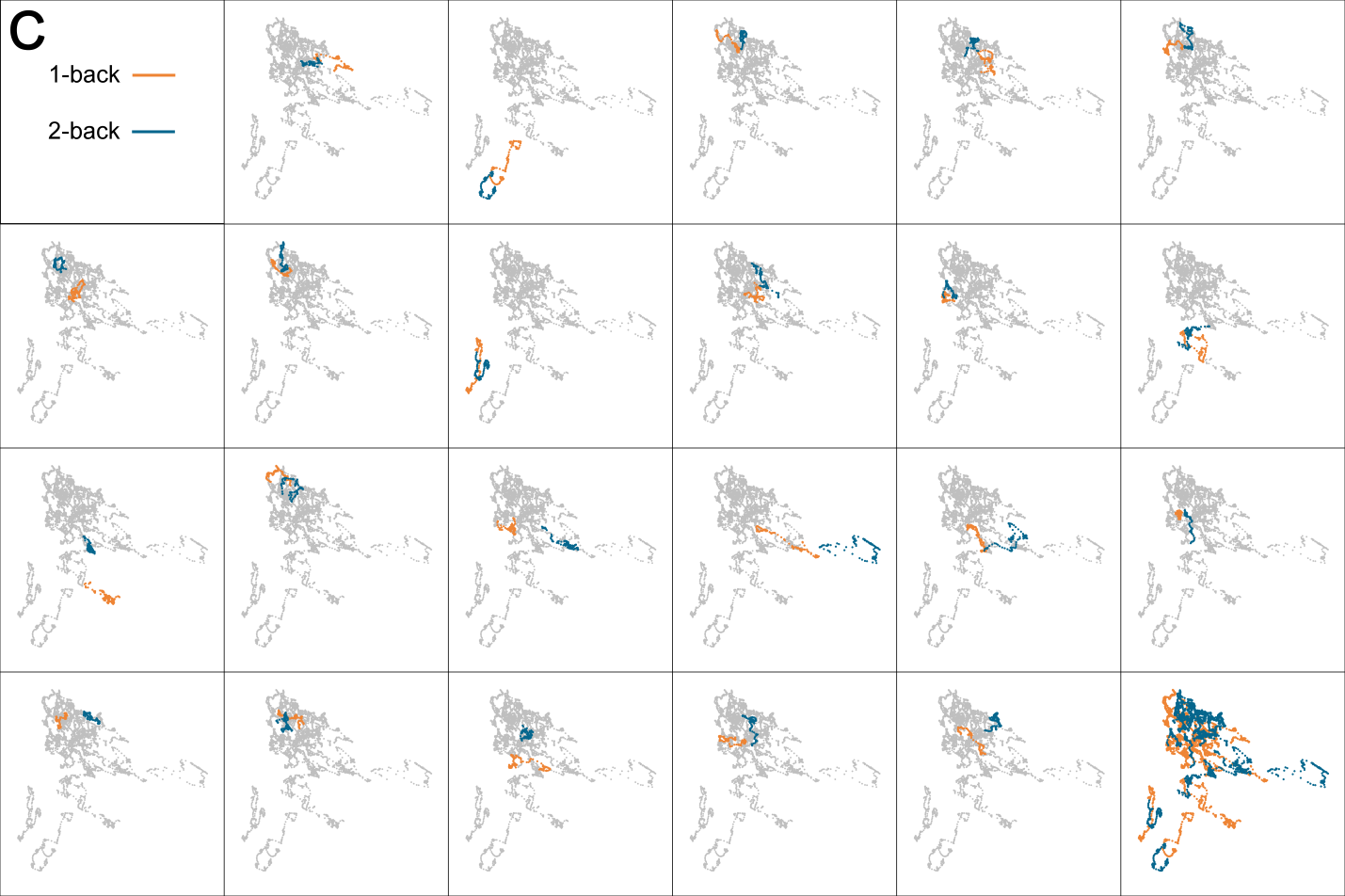 |
| --- |

**Supplemental Figure 3.** **Task-based embedded** **dynamic networks colored by individual participant generated with PCA using the top two components**. Each of the 22 individual participants is represented in a separate panel with their embedded networks colored by task for **(A)** 2-back/rest, **(B)** 1-back/rest, and **(C)** 2-back/1-back. In each of the participant-specific panels all other participants are gray. The bottom right corner of each figure shows all embedded networks colored as shown in the top left corner.

| 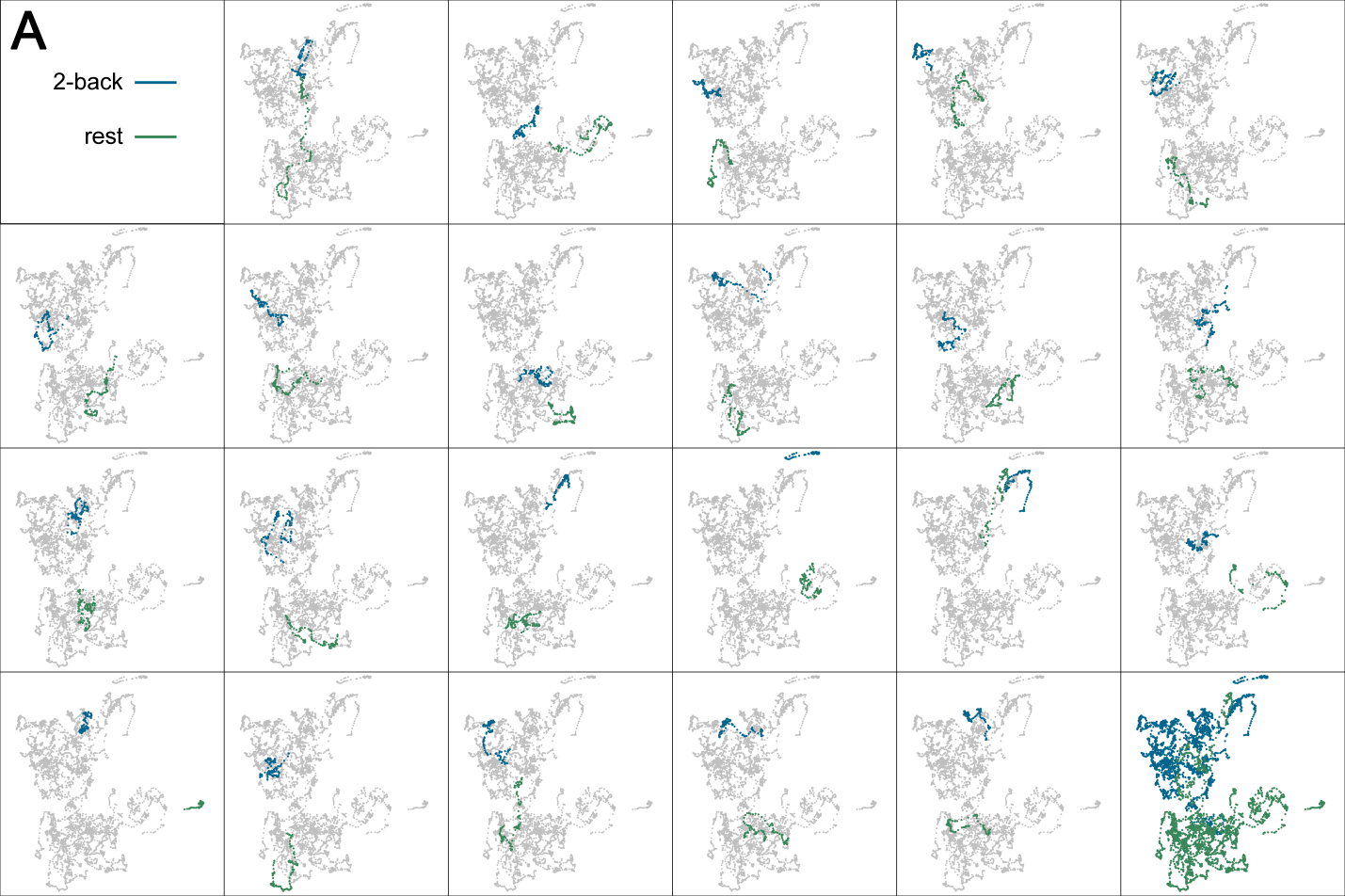 |
| --- |
| 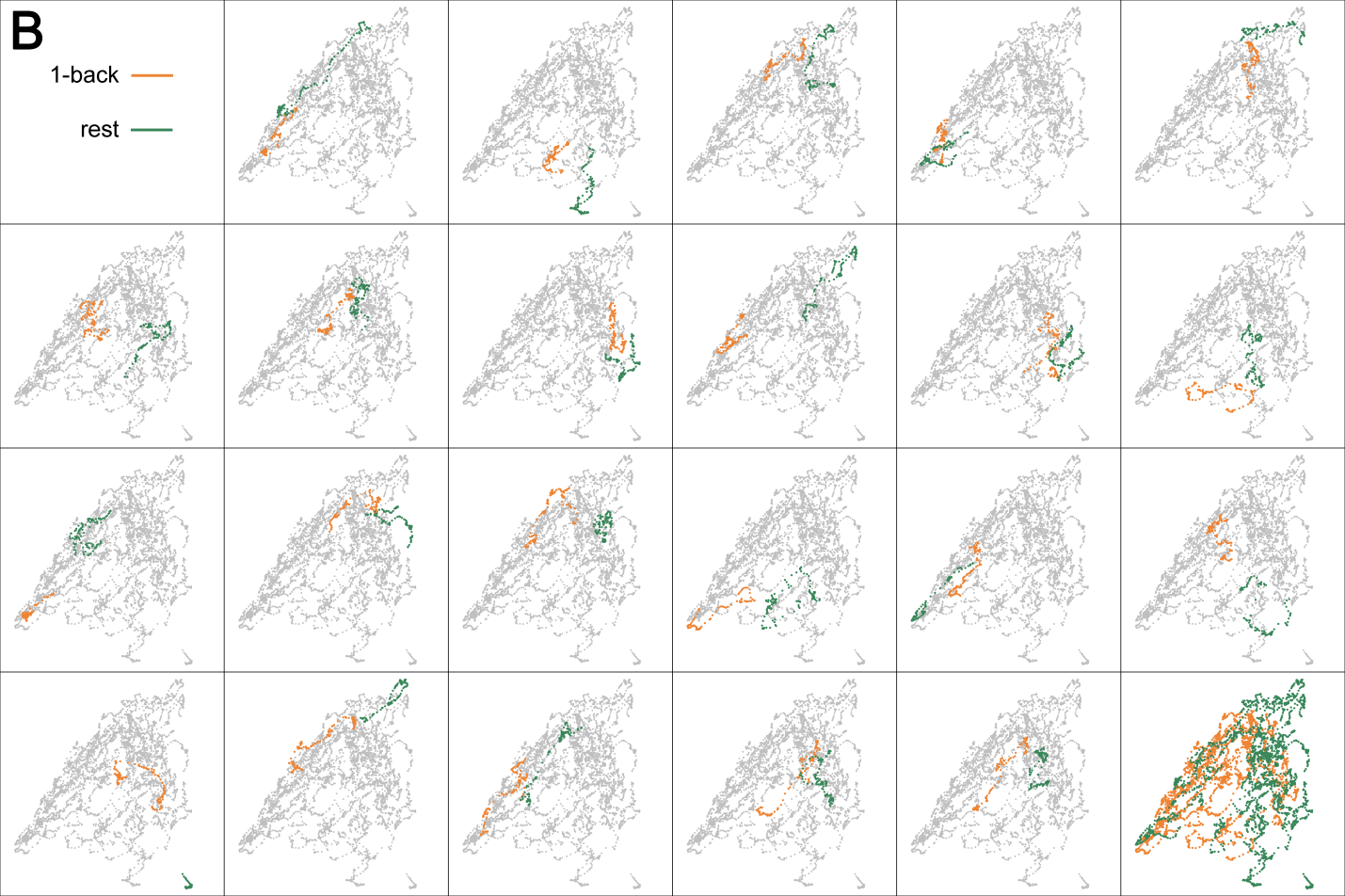 |

| 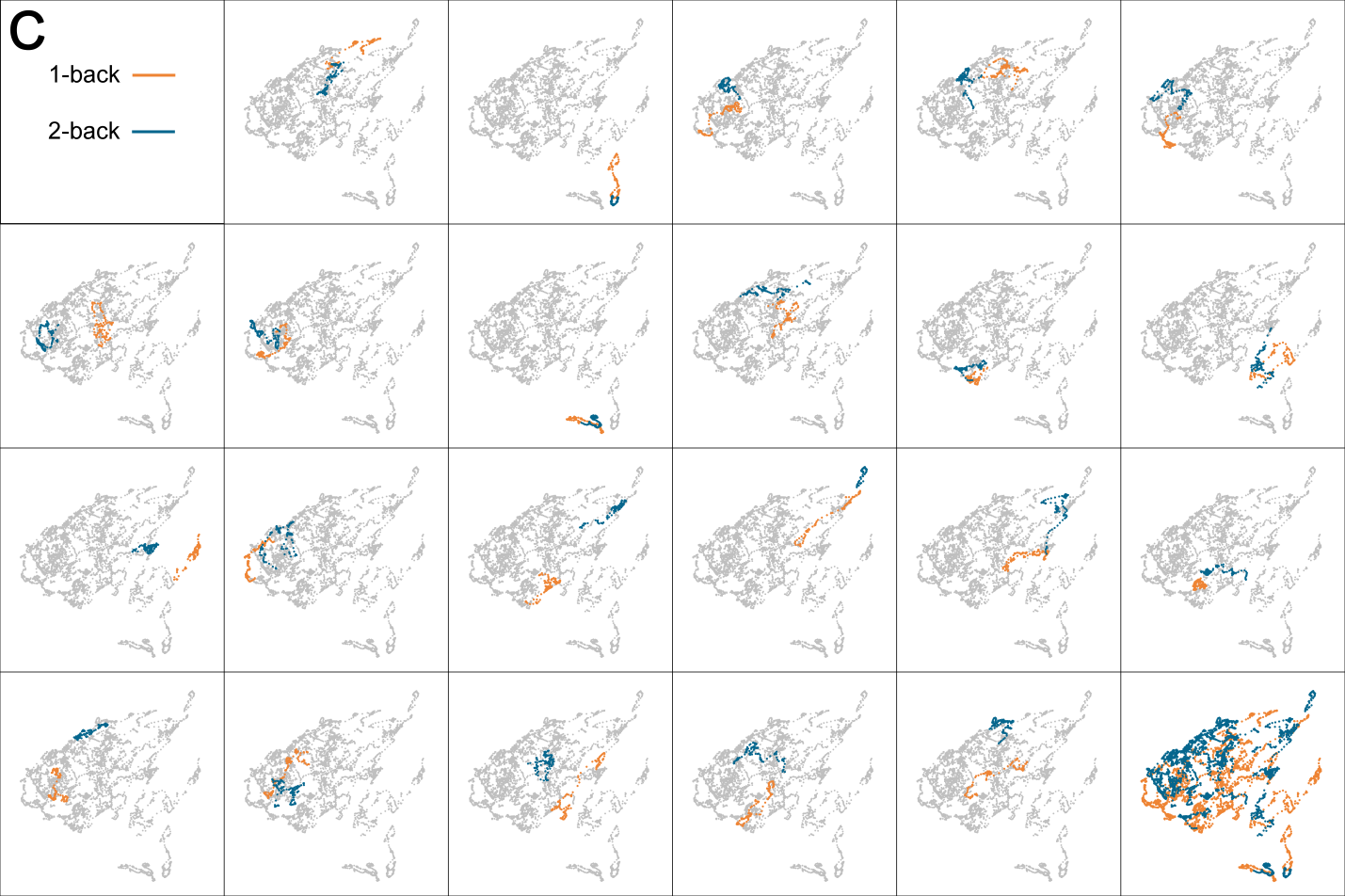 |
| --- |

**Supplemental Figure 4.** **Task-based embedded** **dynamic networks colored by individual participant generated with t-SNE using the top two components**. Each of the 22 individual participants is represented in a separate panel with their embedded networks colored by task for **(A)** 2-back/rest, **(B)** 1-back/rest, and **(C)** 2-back/1-back. In each of the participant-specific panels all other participants are gray. The bottom right corner of each figure shows all embedded networks colored as shown in the top left corner.

| 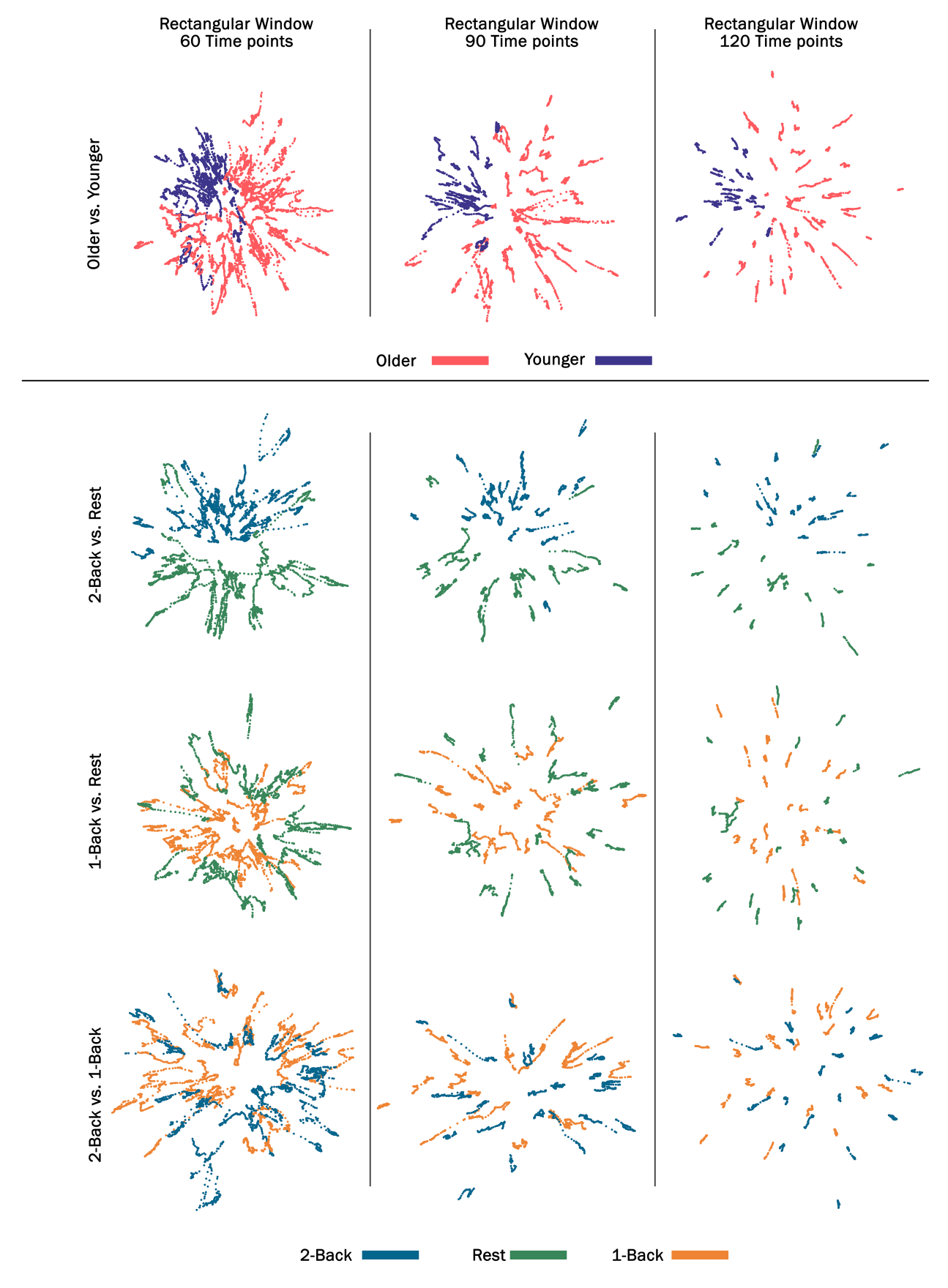  **Supplemental Figure 5.** **Embedded** **dynamic networks generated using varying window lengths**. The first column shows the original embedded data for group and task comparisons. The next two columns show embedded dynamic networks generated using windows with 90 and 120 time points. Note that while specific details necessarily changed since the number of dynamic networks changes with altered window size, the mappings are qualitatively similar for all four comparisons. |
| --- |


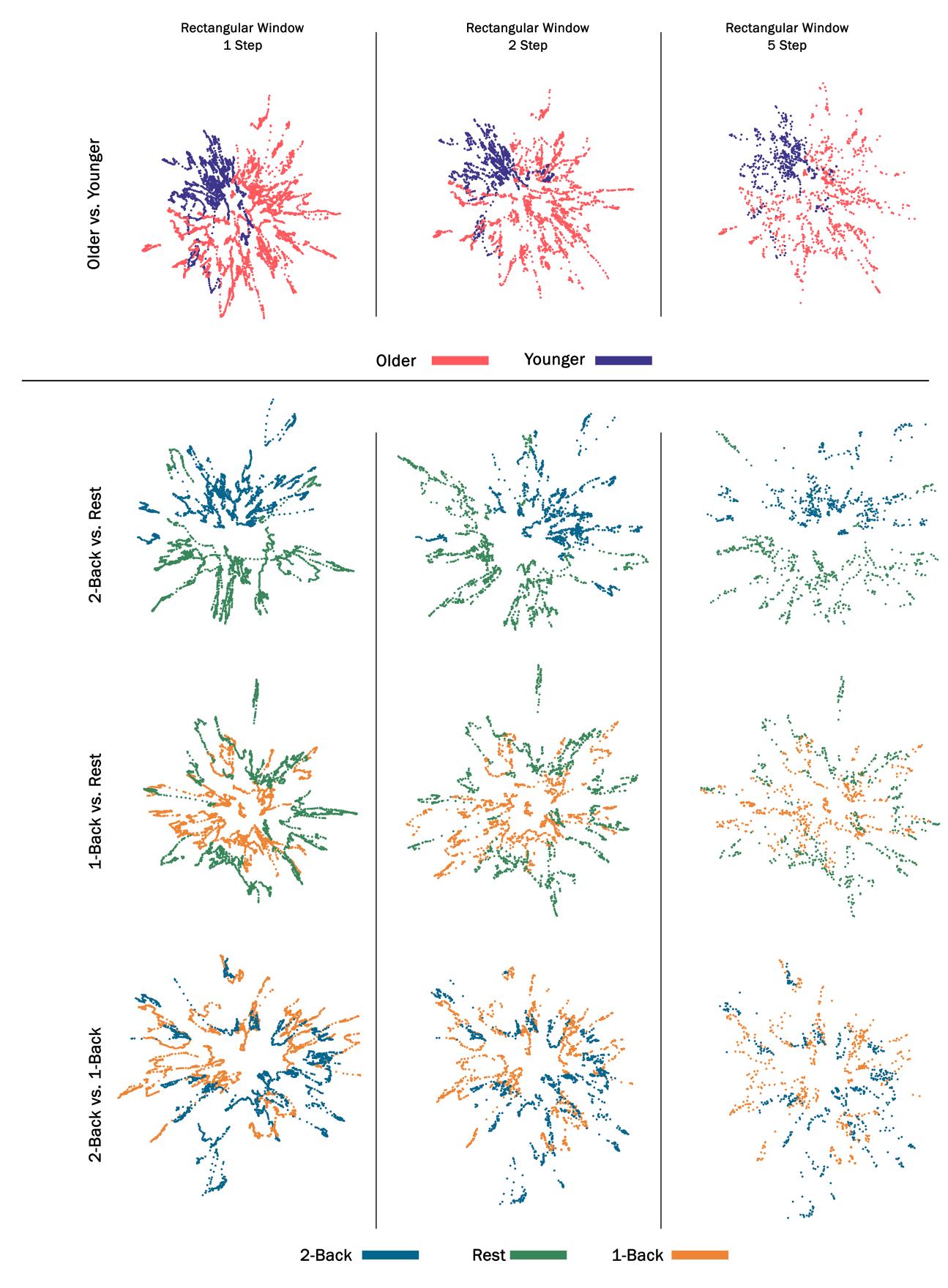


**Supplemental Figure 6.** **Embedded** **dynamic networks generated using varying step sizes**. The first column shows the original embedded data for group and task comparisons using a step size of 1 image volume. The next two columns show embedded dynamic networks generated using step sizes of 2 and 5 image volumes. Note that while specific details necessarily changed since the number of dynamic networks changes with altered step size, the mappings are qualitatively similar for all four comparisons.


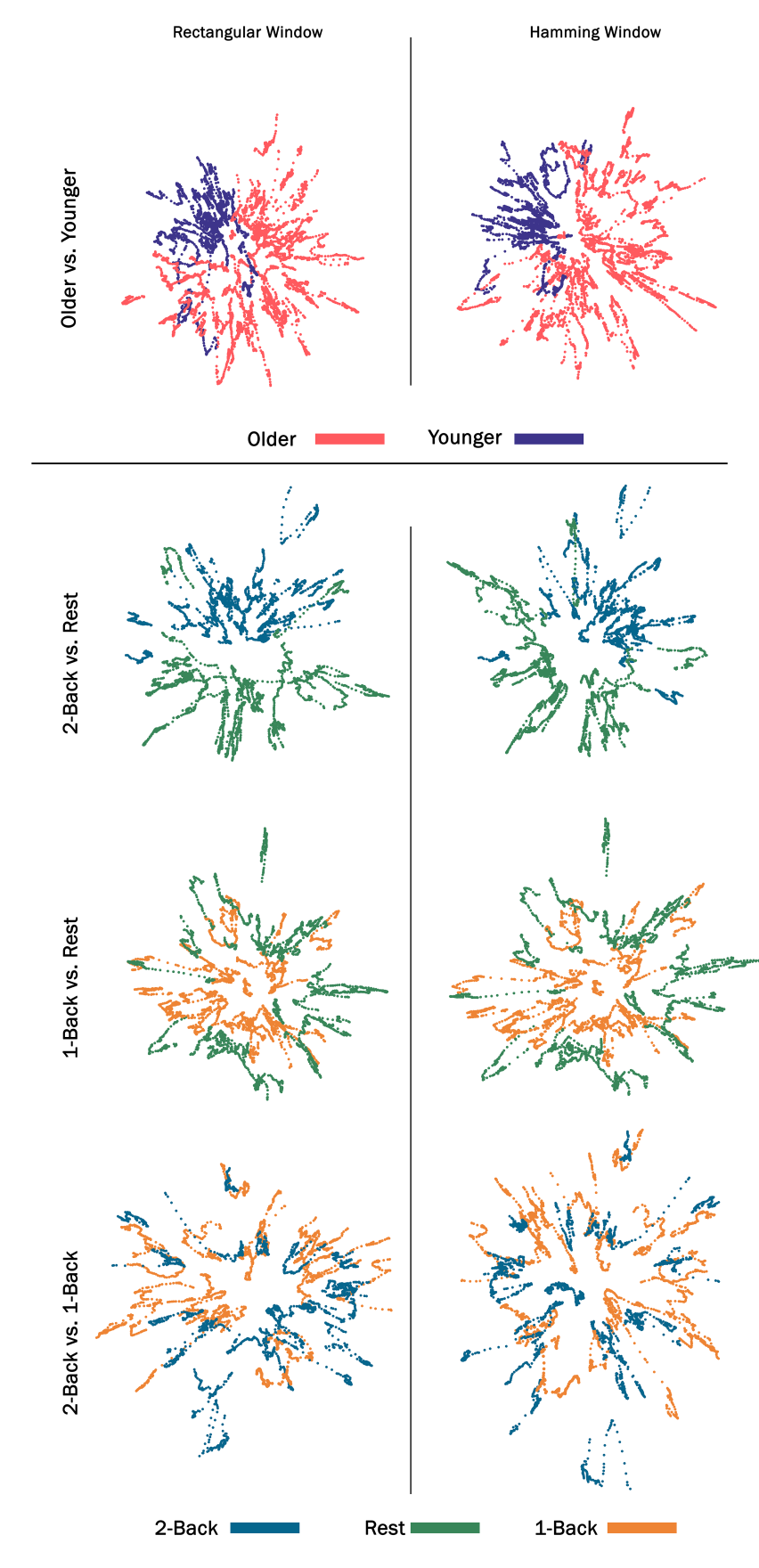


**Supplemental Figure 7.** **Embedded** **dynamic networks generated using rectangle and hamming windows**. The first column shows the original embedded data for group and task comparisons generated using a rectangular window. Column two shows embedded dynamic networks generated using a window weighted with a Hamming function. Although there are subtle differences, the mappings are strikingly similar for all four comparisons.


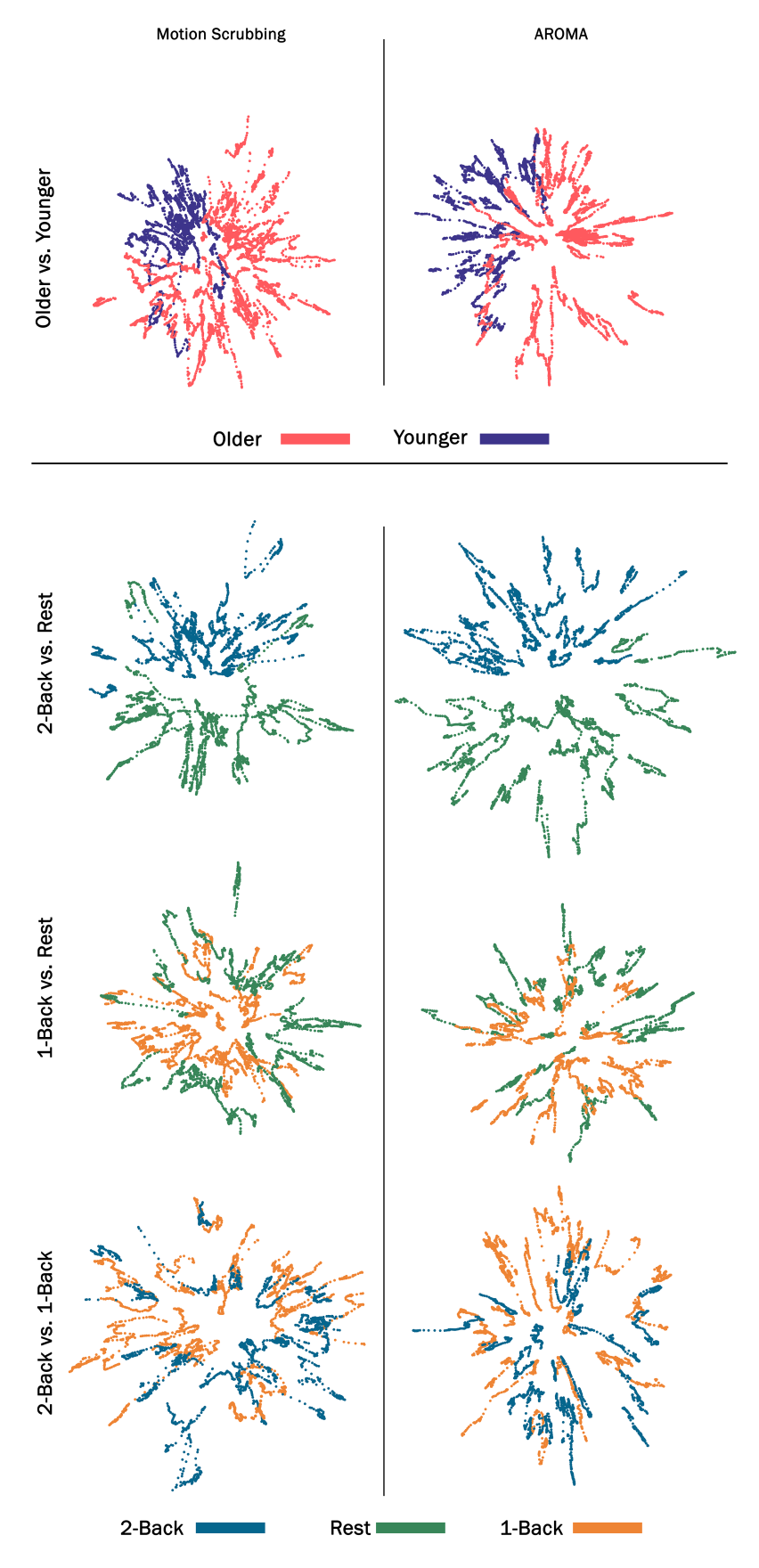


**Supplemental Figure 8.** **Embedded** **dynamic networks generated following different motion correction methods applied to the original functional imaging data.** The first column shows the original embedded data for group and task comparisons generated following the use of the so-called motion scrubbing method that removes (censors) deviant images. Column two shows embedded dynamic networks generated following the use of AROMA for motion correction. This method does not remove deviant image volumes. Although the main conclusions from these figures are consistent, there appear to be differences that are greater than observed when manipulating the window parameters. Future work is necessary to quantify the effects of these and other motion correction methods on the embedded dynamic networks. See methods for motion correction details.


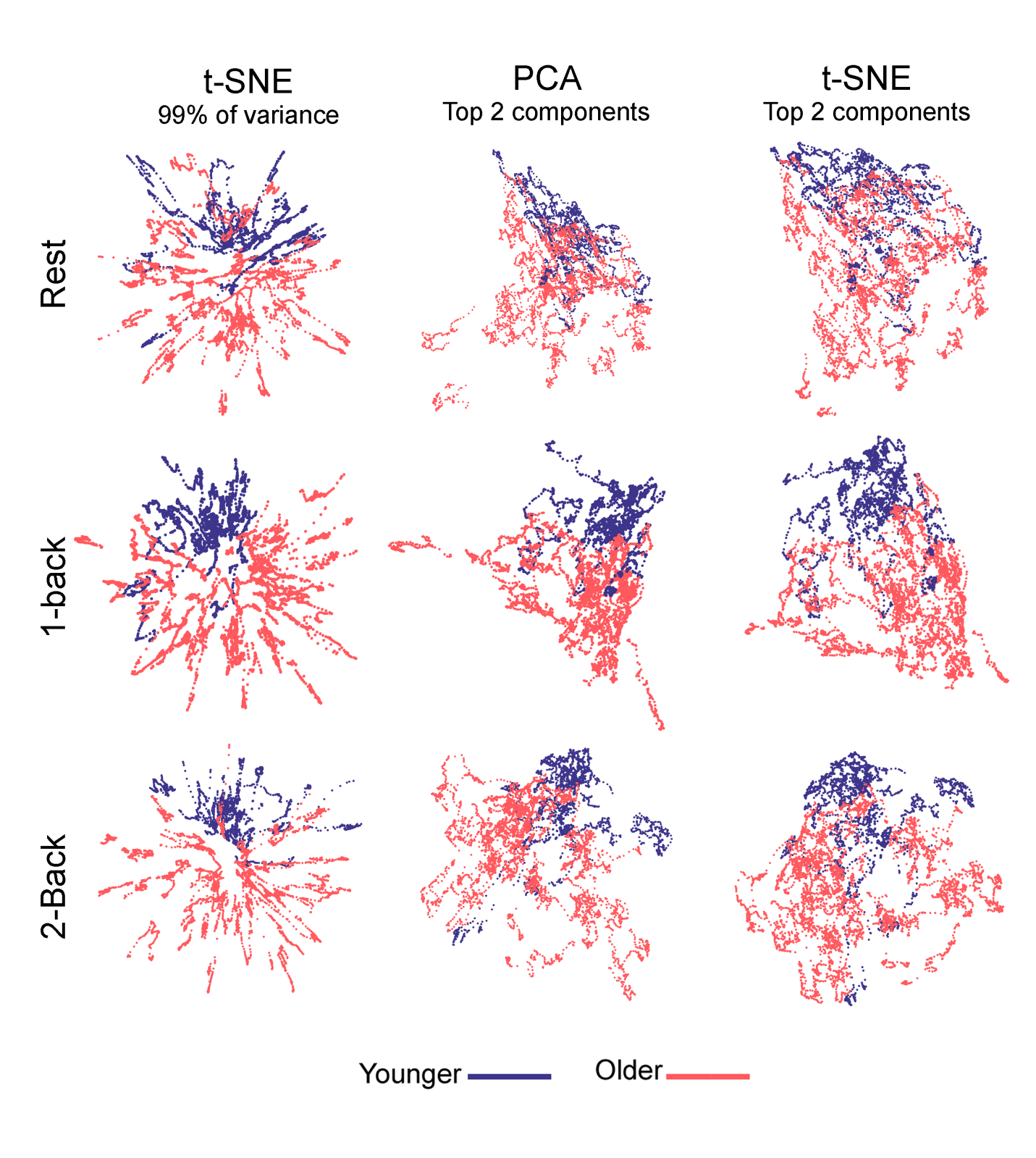


**Supplemental Figure 9.** **Embedded dynamic networks across groups for all three conditions.** Embedded dynamic networks are shown for younger and older adults during the rest, 1-back, and 2-back working memory tasks. The embedded dynamic networks for 1-back task are the same as the ones shown in figure 3 (here, they are shown again for comparison purposes). The group maps made with t-SNE using 99% of the variance is distinct from the other two maps for all three conditions. For 1-back and 2-back working memory tasks, the two groups are visibly distinct for almost all three embedding methods, with embedded networks with t-SNE using 99% of the variance being more distinct for the 2-back working memory task. For the rest, the overlap between the embedded networks for the younger and older adults increases.


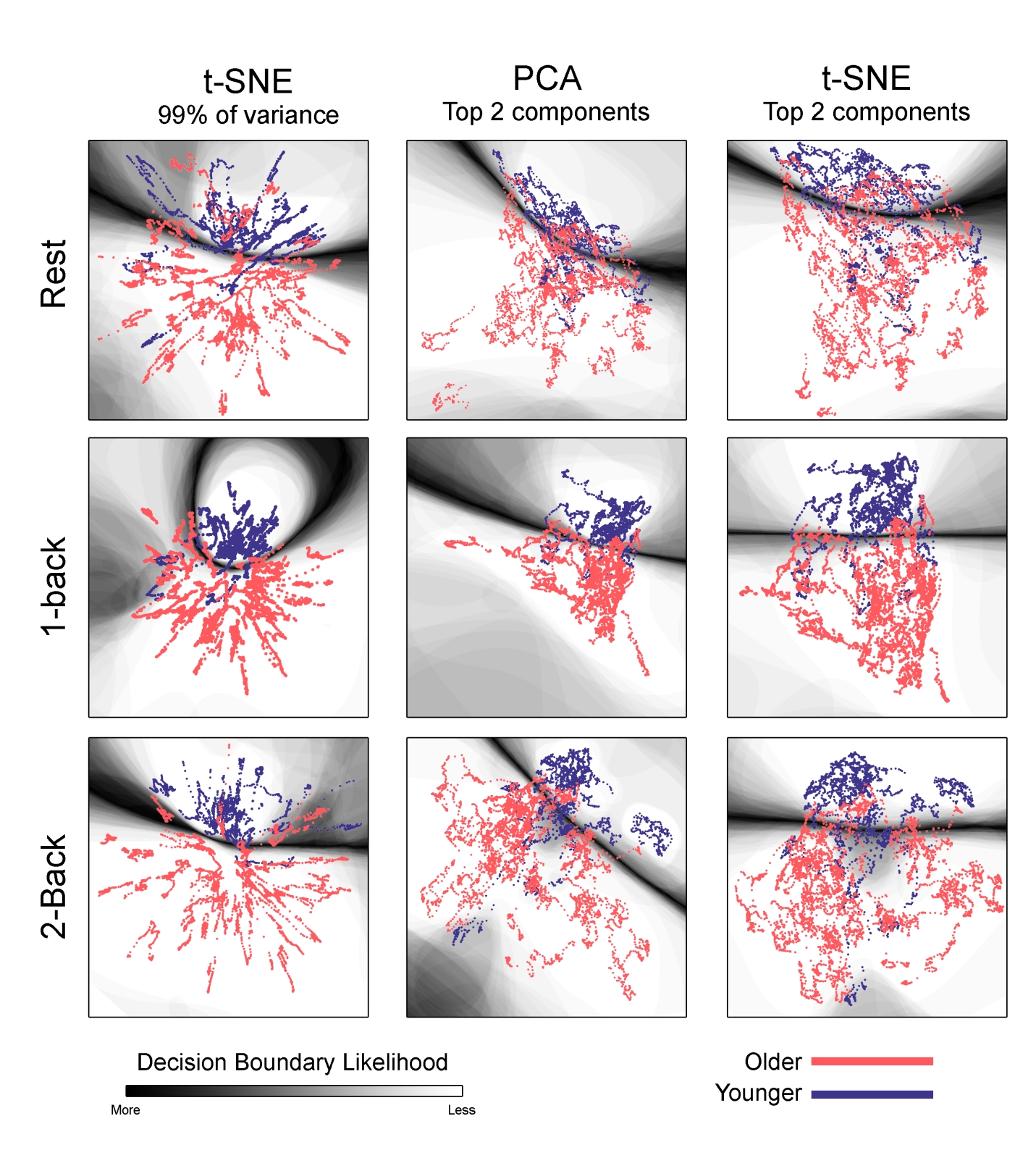


**Supplemental Figure 10.** **Classification boundaries for embedded group-based networks for all three conditions.** Boundaries for classifying younger vs. older adults during the rest, 1-back, and 2-back working memory tasks are shown here. The boundaries shown for the 1-back task are the same as the ones shown in figure 6 (here, they are shown again for comparison purposes). Embedded methods are labeled above each mapping. For the 1-back and 2-back working memory tasks, for all embedding methods the boundary is fairly distinct and separates the younger and older adults. For the rest, the boundary is less distinct. See figure 5 of the paper for more detail.

|  | Distance | Std Dev |
| --- | --- | --- |
|  | **t-SNE with 99% of variance** | |
| 2-Back vs Rest | 143.66 | 61.02 |
| 1-Back vs Rest | 92.82 | 46.76 |
| 2-Back vs 1-Back | 72.76 | 42.54 |
|  | **PCA with top 2 components** | |
| 2-Back vs Rest | 103.91 | 47.37 |
| 1-Back vs Rest | 80.39 | 53.27 |
| 2-Back vs 1-Back | 60.49 | 35.09 |
|  | **t-SNE with top 2 components** | |
| 2-Back vs Rest | 179.95 | 56.33 |
| 1-Back vs Rest | 114.89 | 51.46 |
| 2-Back vs 1-Back | 83.24 | 38.05 |
| Supplemental Table 1. Distance between task-based embedded networks averaged across participants. The distance (in arbitrary units) between the two embedded networks was computed for each of the task comparisons and embedding methods in every participant. The average and standard deviation across the participants are presented. | | |

|  | F value | p-value |
| --- | --- | --- |
|  | **t-SNE with 99% of variance** | |
| F-test | 13.79 | <0.001 |
|  | *Post-hoc comparisons* | |
| 2B/R vs 1B/R | 19.85 | <0.001 |
| 2B/R vs 2B/1B | 29.91 | <0.001 |
| 1B/R vs 2B/1B | 3.06 | 0.095 |
|  | **PCA with top 2 components** | |
| F-test | 16.73 | <0.001 |
|  | *Post-hoc comparisons* | |
| 2B/R vs 1B/R | 9.55 | 0.006 |
| 2B/R vs 2B/1B | 26.61 | <0.001 |
| 1B/R vs 2B/1B | 2.81 | 0.109 |
|  | **t-SNE with top 2 components** | |
| F-test | 46.00 | <0.001 |
|  | *Post-hoc comparisons* | |
| 2B/R vs 1B/R | 38.56 | <0.001 |
| 2B/R vs 2B/1B | 97.27 | <.0001 |
| 1B/R vs 2B/1B | 6.96 | 0.0154 |
| Supplemental Table 2. Statistical comparison of distance between task-based embedded networks. A MANOVA was used to compare the distance between embedded networks for each task comparison and embedding method. Post-hoc contrasts were used to identify significant paired comparisons. Abbreviations: 2B= 2-back, 1B=1-back, R=rest. | | |
